# Supplementary material for: How J-chain ensures the assembly of immunoglobulin IgM pentamers
Source: EMBO J. 2024 Dec 4;44(2):505–33. doi: 10.1038/s44318-024-00317-9 (PMC11729874; doi:10.1038/s44318-024-00317-9)
Supplement: Supplementary file 1 — Appendix [file 44318_2024_317_MOESM1_ESM.pdf]

Appendix

How J-chain ensures the assembly of immunoglobulin IgM pentamers

Chiara Giannone, Xenia Mess, Ruiming He, Maria Rita Chelazzi, Annika Mayer, Anush Bakunts, Tuan Nguyen, Yevheniia Bushman, Andrea Orsi, Benedikt Gansen, Massimo Degano, Johannes Buchner*, and Roberto Sitia*

* corresponding authors

Table of Contents

Appendix Figure S1: Sequence conservation of JC during evolution **3**

Appendix Figure S2: Quality control of recombinant proteins... **4**

Appendix Figure S3: Organization of the µtps in μ_2L_2 subunits of pentameric IgM **5**

.

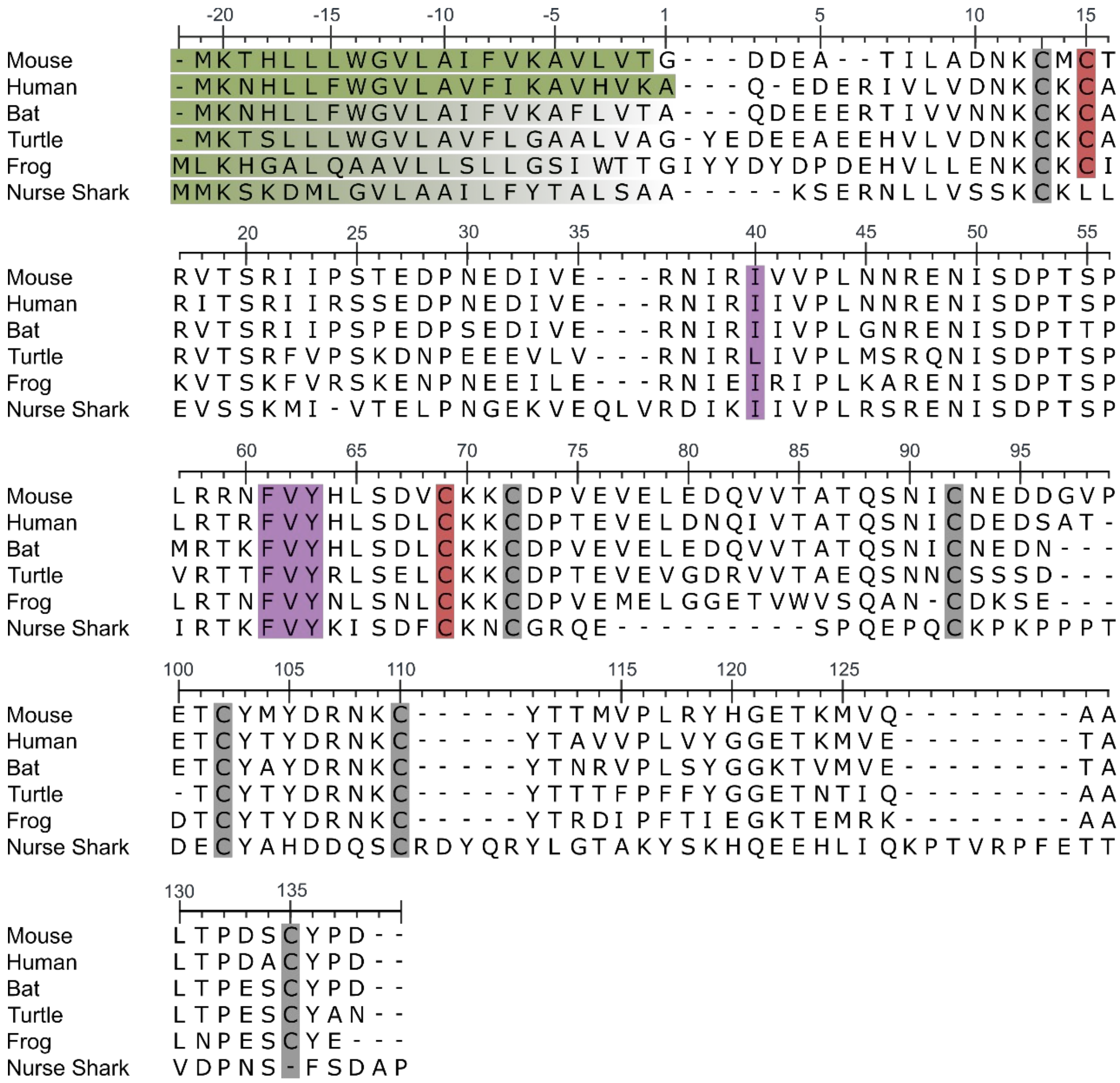

Appendix Figure S1 - Sequence conservation of JC during evolution.

From their first appearance in nurse sharks, JC remain largely conserved, a surprising feature for an intrinsically disordered polypeptide that folds only upon interaction with nascent IgM pentamers. Virtually unchanged are I40, F61, Y63 (purple) and, the eight cysteines forming intramolecular cysteine bridges (grey) or intermolecular bonds with IgM (red). The sequences responsible for importing proteins into the endoplasmic reticulum (ER) are denoted by a green color and are not considered part of the sequence numbering.

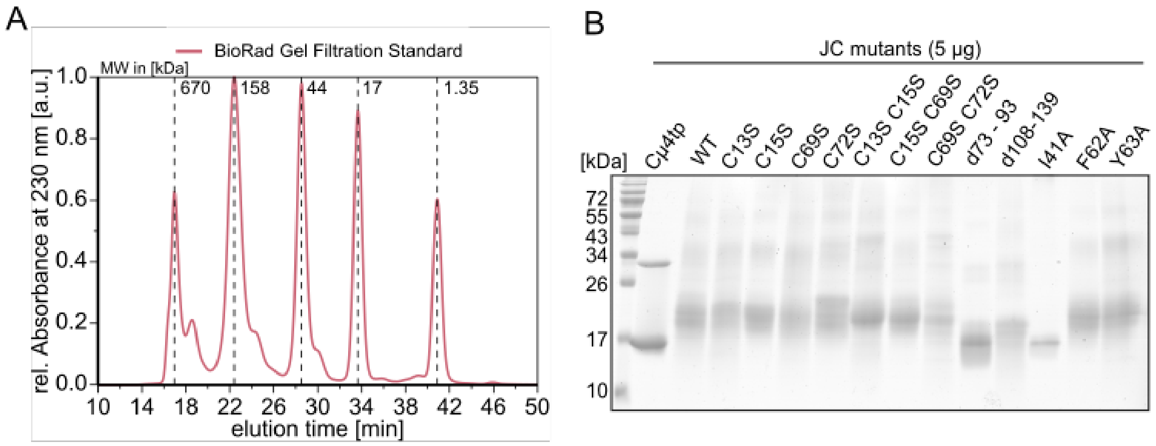

Appendix Figure S2 - Quality control of recombinant proteins

Panel **A**: Chromatogram of molecular weight standards: Thyroglobulin (bovine): 670 kDa, γ-globulin (bovine): 158 kDa, ovalbumin (chicken): 44 kDa, myoglobin (horse): 17 kDa, vitamin B12: 1.350 kDa.

Panel **B**: JC is predominantly monomeric on non-reducing SDS-PAGE.

Most recombinant JC forms monomers after refolding and oxidation, which are visible as smear with an apparent mass of > 17 kDa on non-reducing SDS-PAGE. Covalently bridged species form small amounts of oligomers visible above the monomer bands. Cµ4tp is present as monomers (< 17 kDa), which can be oxidized to dimers (< 34 kDa) in the absence of reducing agents like DTT.

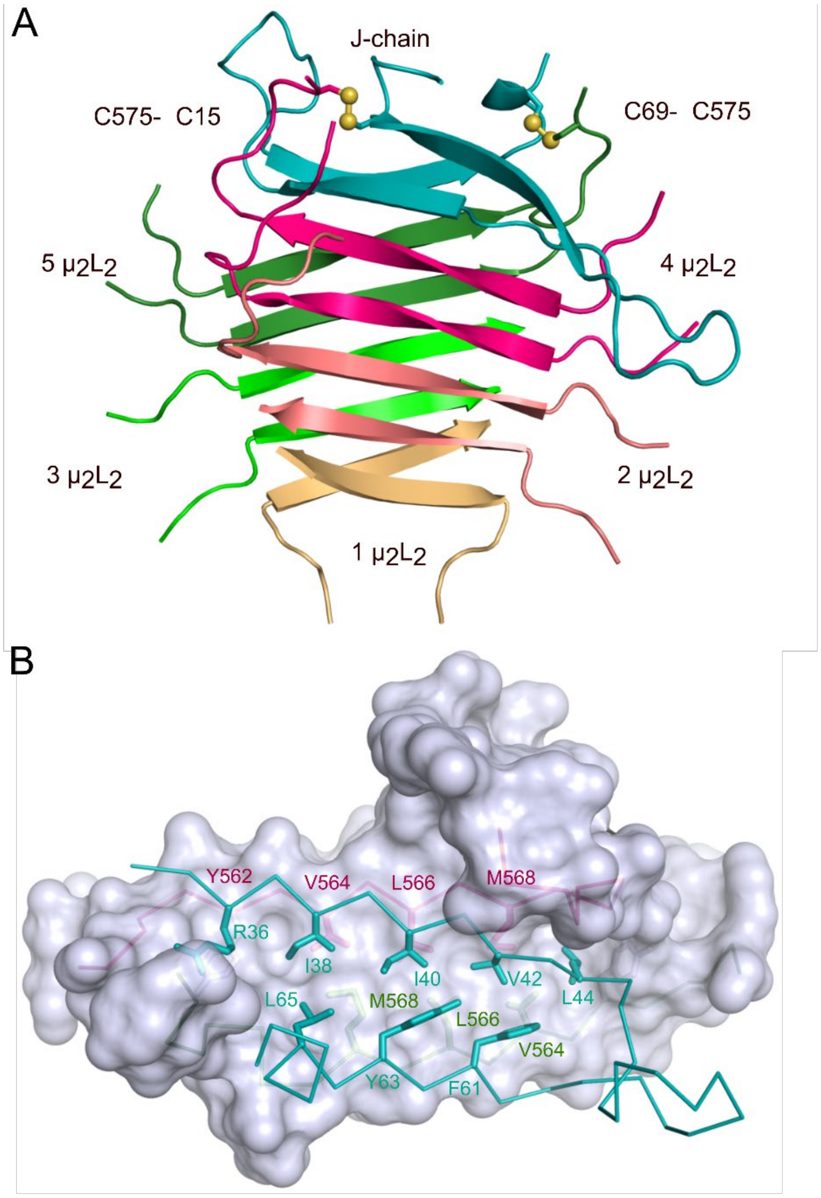

Appendix Figure S3 - Organization of the µtps in μ_2L_2 subunits of pentameric IgM.

Panel **A** shows a front view of the core of pentameric IgM, with the two disulfide bonds between JC and C575 highlighted as yellow spheres). Note the opposite µtp arrangements in subunit 1 (in orange, bottom part of the figure) compared to the other four. Note also how the two beta-strands from the J chain are mimicking the µtps arrangements in the pentamer core, extending the β-sandwich structure**.**

Panel **B** highlights the different arrangements of the µtps that interface with JC.

The protein surface formed by subunits 4 and 5 upon interaction with a JC is portrayed in grey. The images were generated using PyMOL (The PyMOL Molecular Graphics System) are extrapolated from the Cryo-EM structure of human IgM-Fc in complex with the J chain and the ectodomain of pIgR.
