# Supplementary figures and images for: How J-chain ensures the assembly of immunoglobulin IgM pentamers
Source: EMBO J. 2024 Dec 4;44(2):505–33. doi: 10.1038/s44318-024-00317-9 (PMC11729874; doi:10.1038/s44318-024-00317-9)

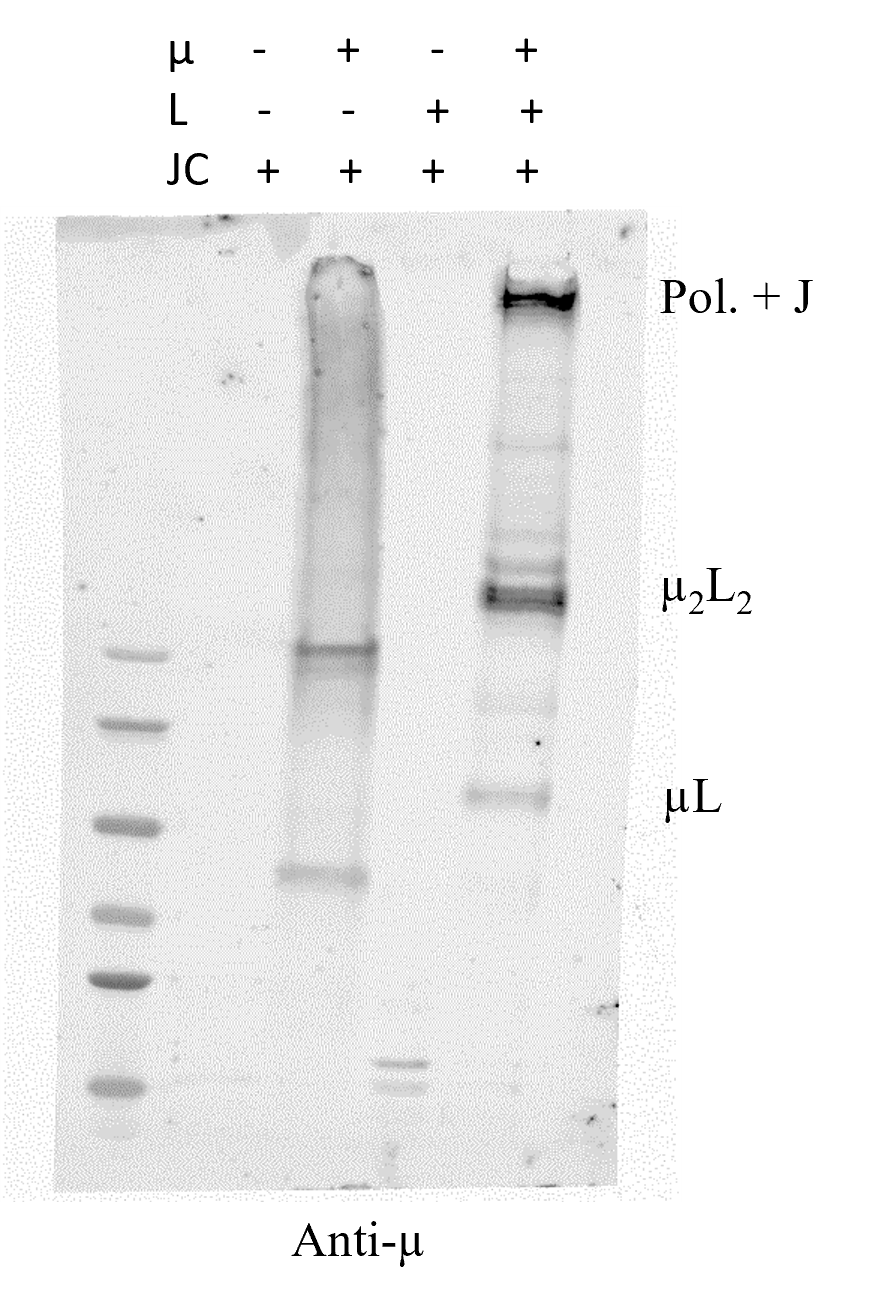

Supplement: Supplementary file 3 — Source data Fig. 1 [file 44318_2024_317_MOESM3_ESM.zip › Figure 1/1C/western heavy chain.tif]

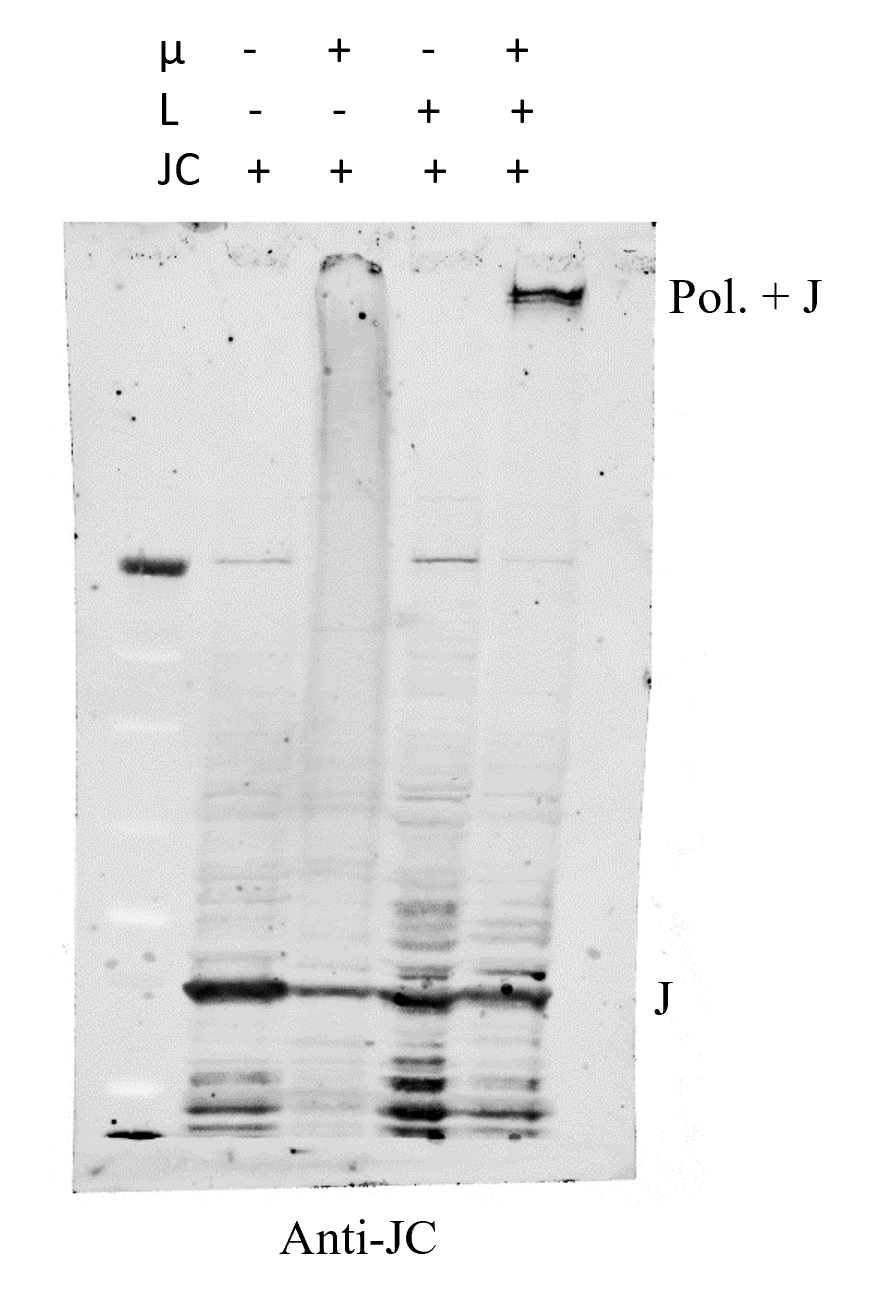

Supplement: Supplementary file 3 — Source data Fig. 1 [file 44318_2024_317_MOESM3_ESM.zip › Figure 1/1C/western JChain.tif]

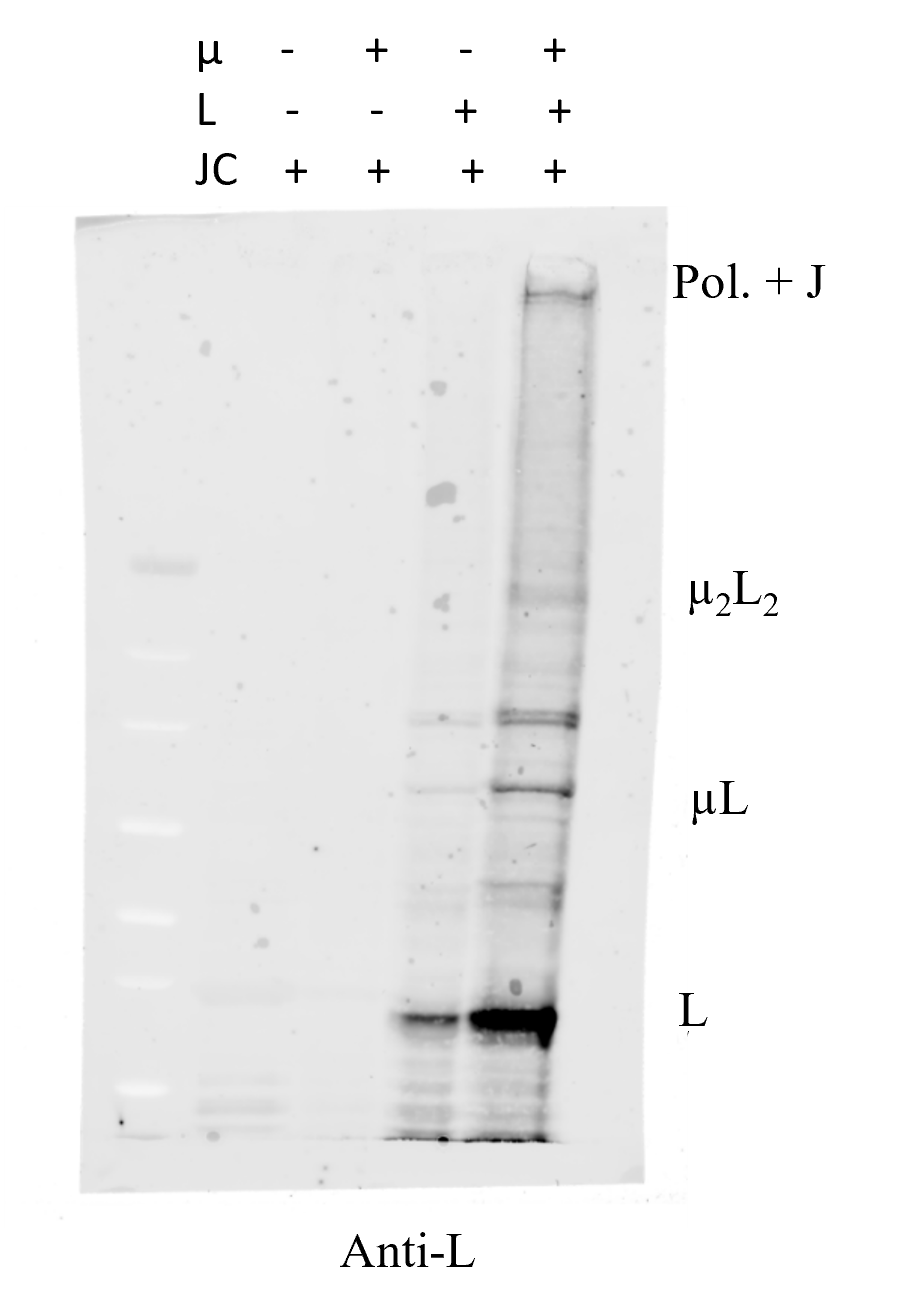

Supplement: Supplementary file 3 — Source data Fig. 1 [file 44318_2024_317_MOESM3_ESM.zip › Figure 1/1C/western Lambda.tif]

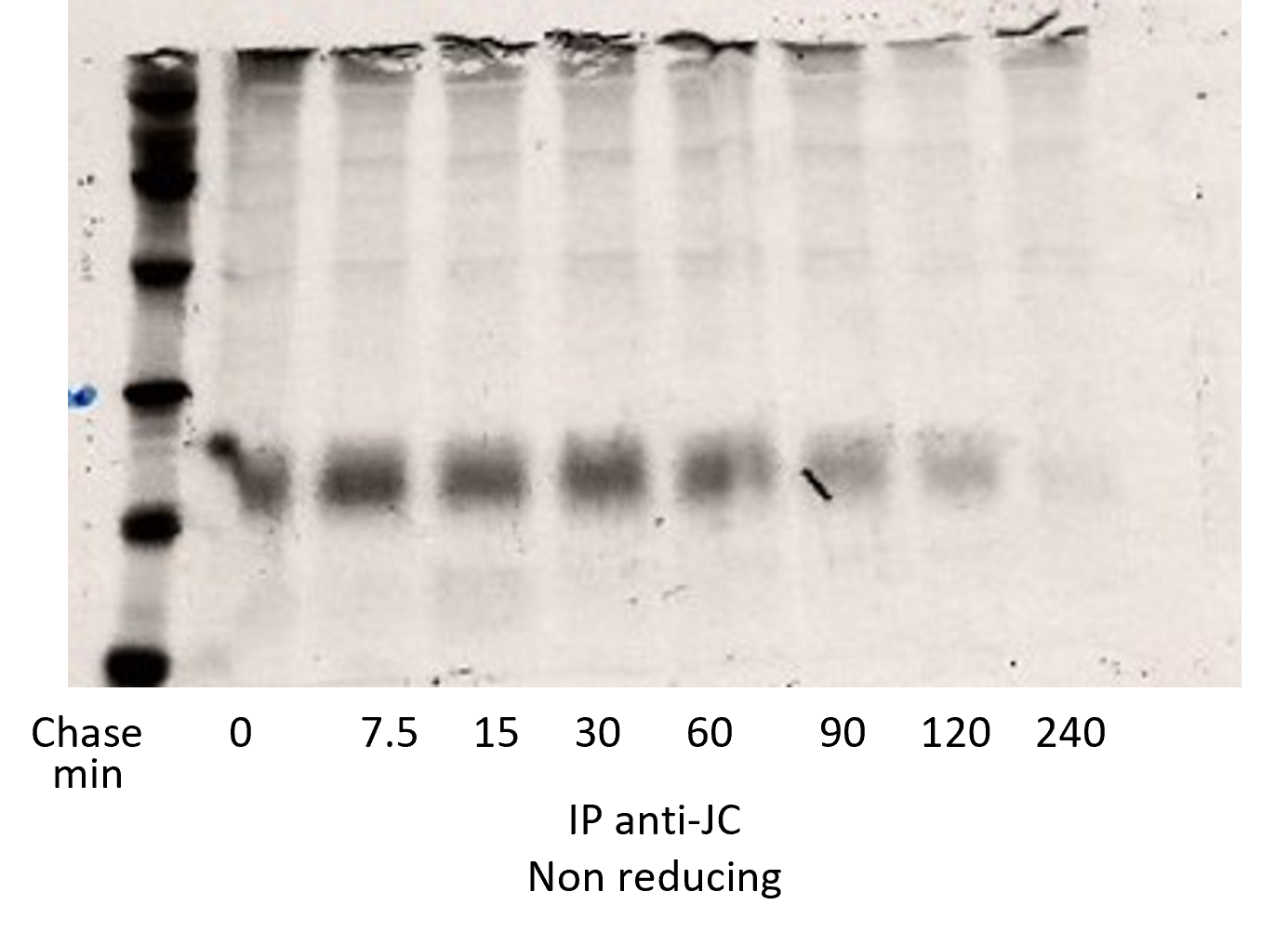

Supplement: Supplementary file 3 — Source data Fig. 1 [file 44318_2024_317_MOESM3_ESM.zip › Figure 1/1D/pulse chase non reducing JC.tif]

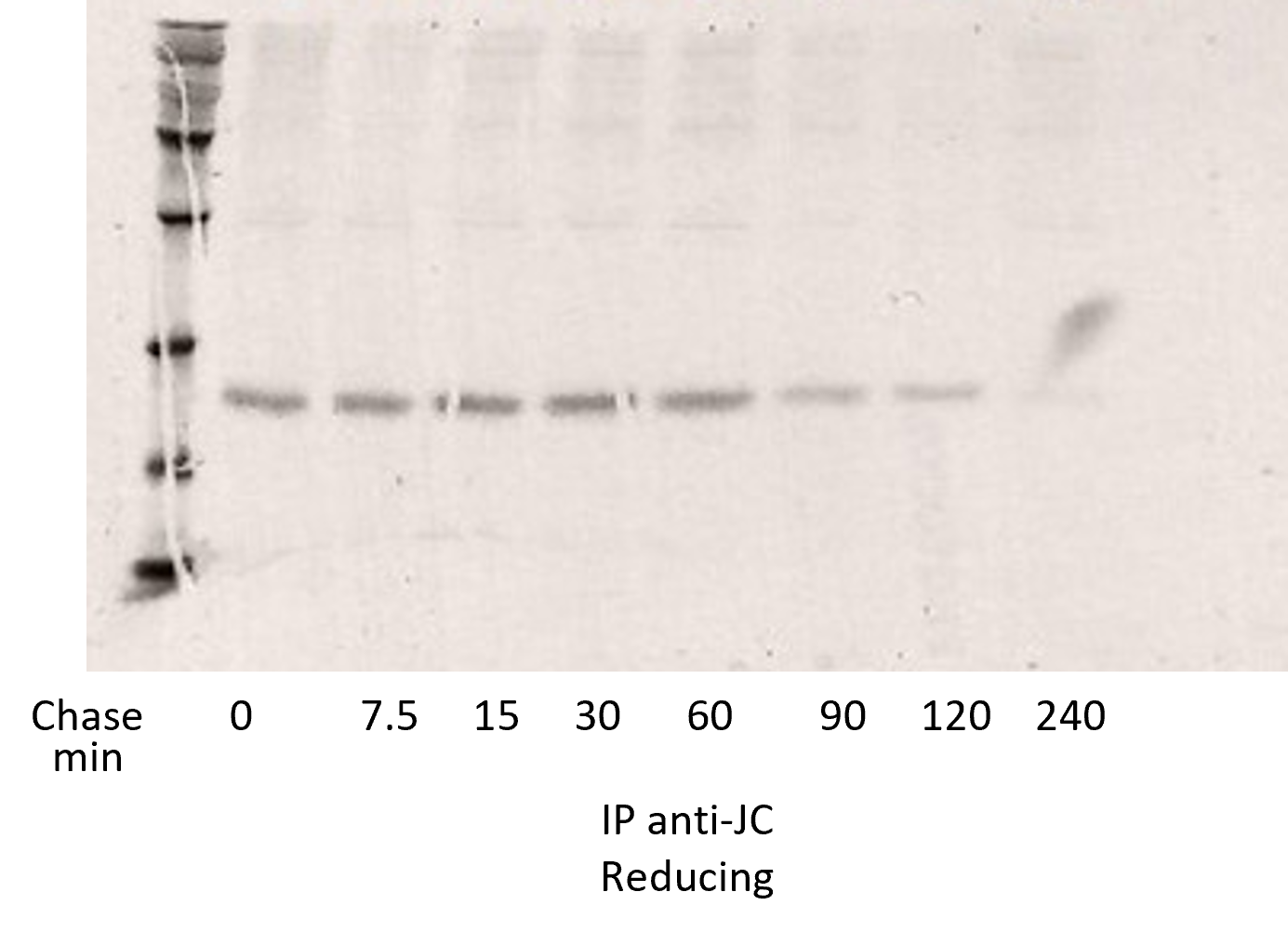

Supplement: Supplementary file 3 — Source data Fig. 1 [file 44318_2024_317_MOESM3_ESM.zip › Figure 1/1D/pulse chase reducing JC.tif]

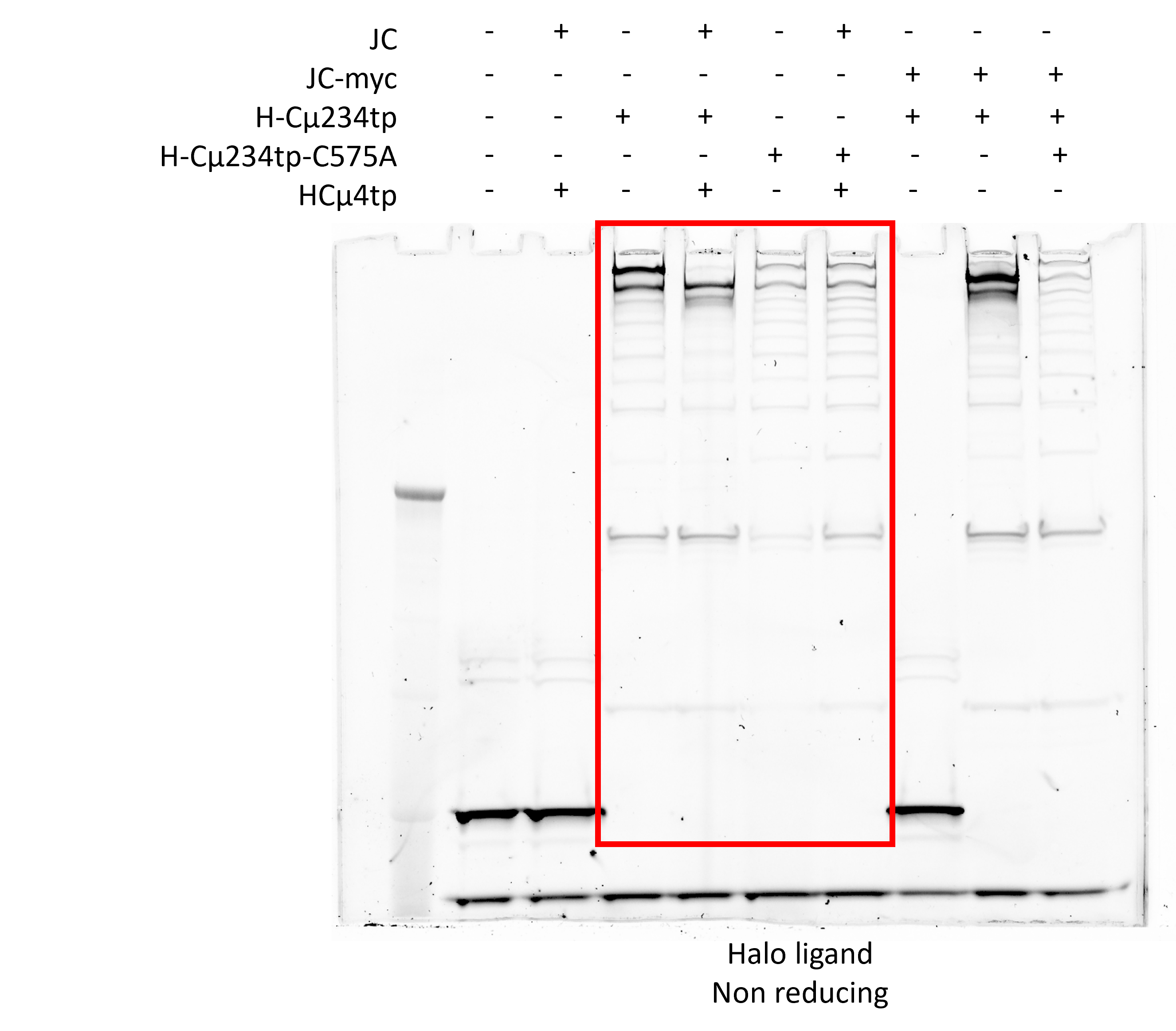

Supplement: Supplementary file 4 — Source data Fig. 2 [file 44318_2024_317_MOESM4_ESM.zip › Figure 2/2A/Halo ligand.tif]

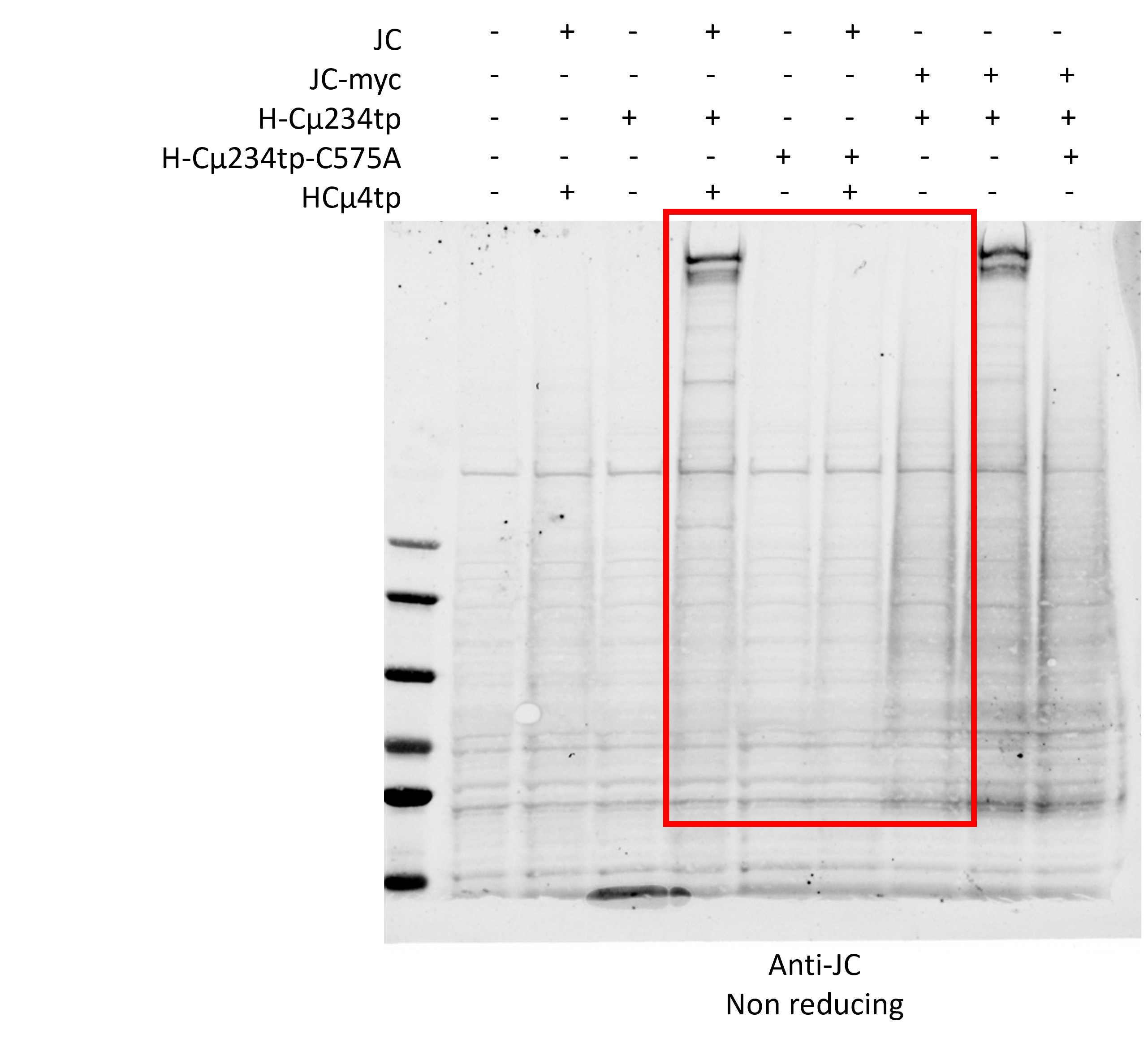

Supplement: Supplementary file 4 — Source data Fig. 2 [file 44318_2024_317_MOESM4_ESM.zip › Figure 2/2A/western JC.tif]

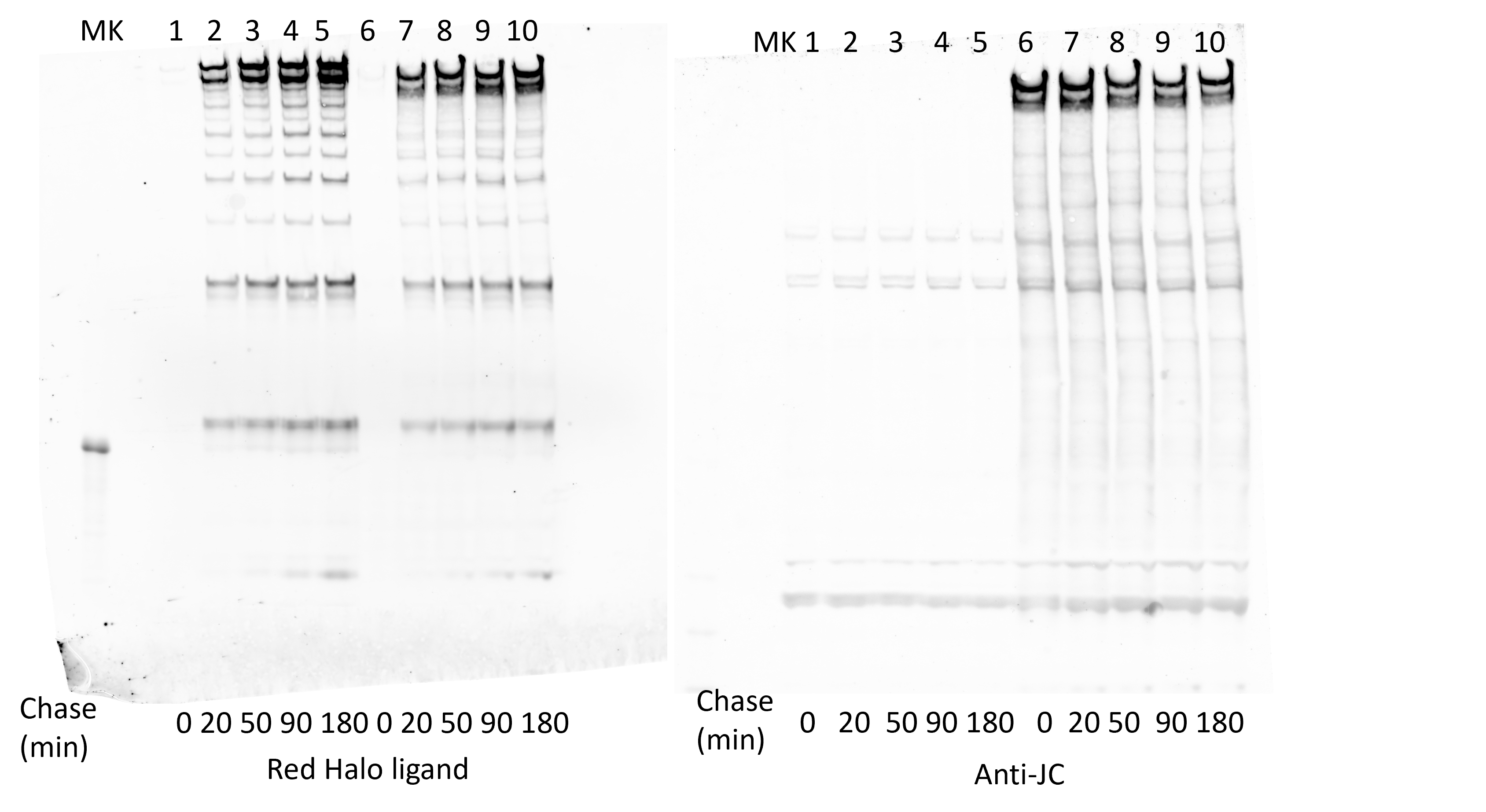

Supplement: Supplementary file 4 — Source data Fig. 2 [file 44318_2024_317_MOESM4_ESM.zip › Figure 2/2B/2B.tif]

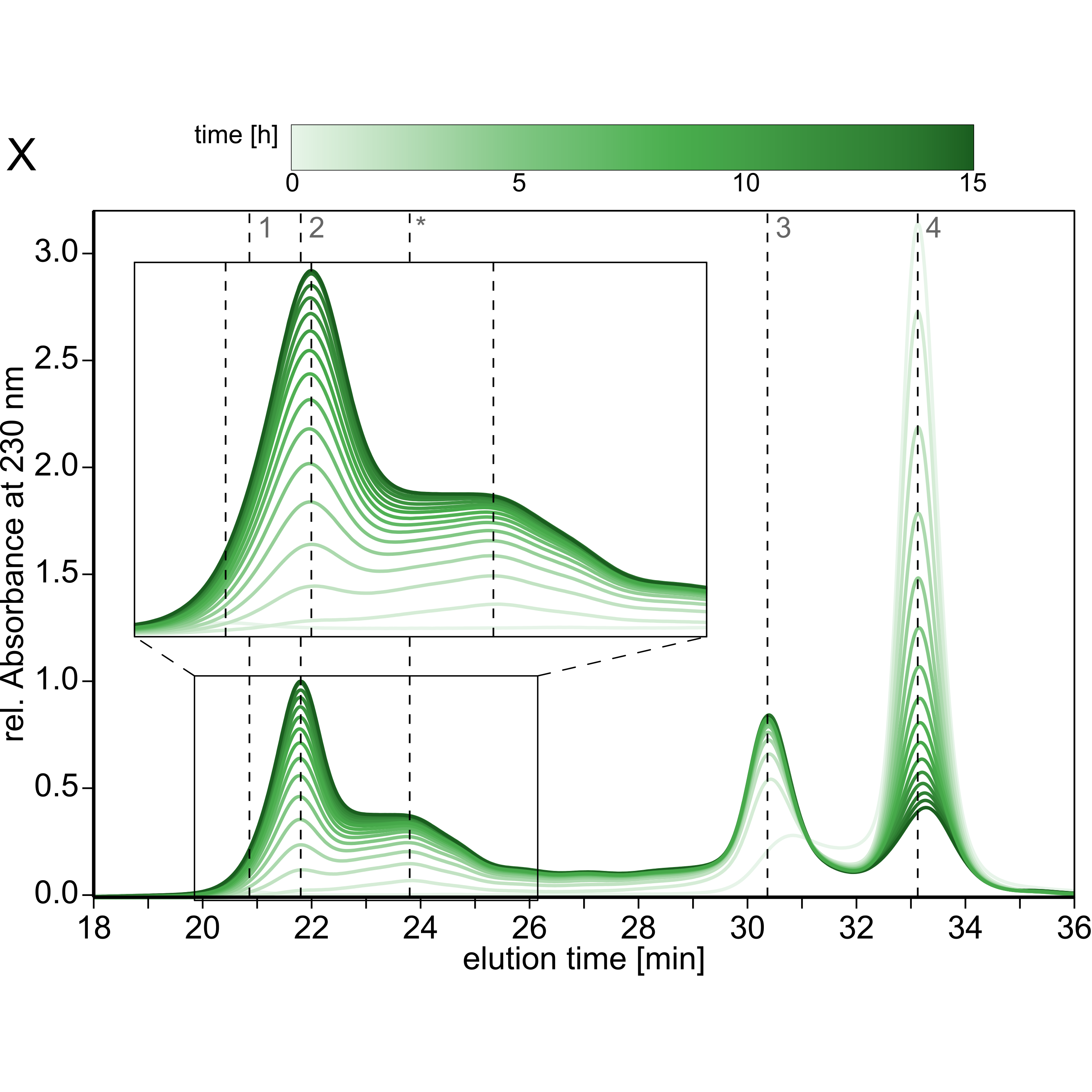

Supplement: Supplementary file 5 — Source data Fig. 3 [file 44318_2024_317_MOESM5_ESM.zip › Figure 3/3B/repeat 1 Cu4 + JC/Kinetics_1 with insert.png]

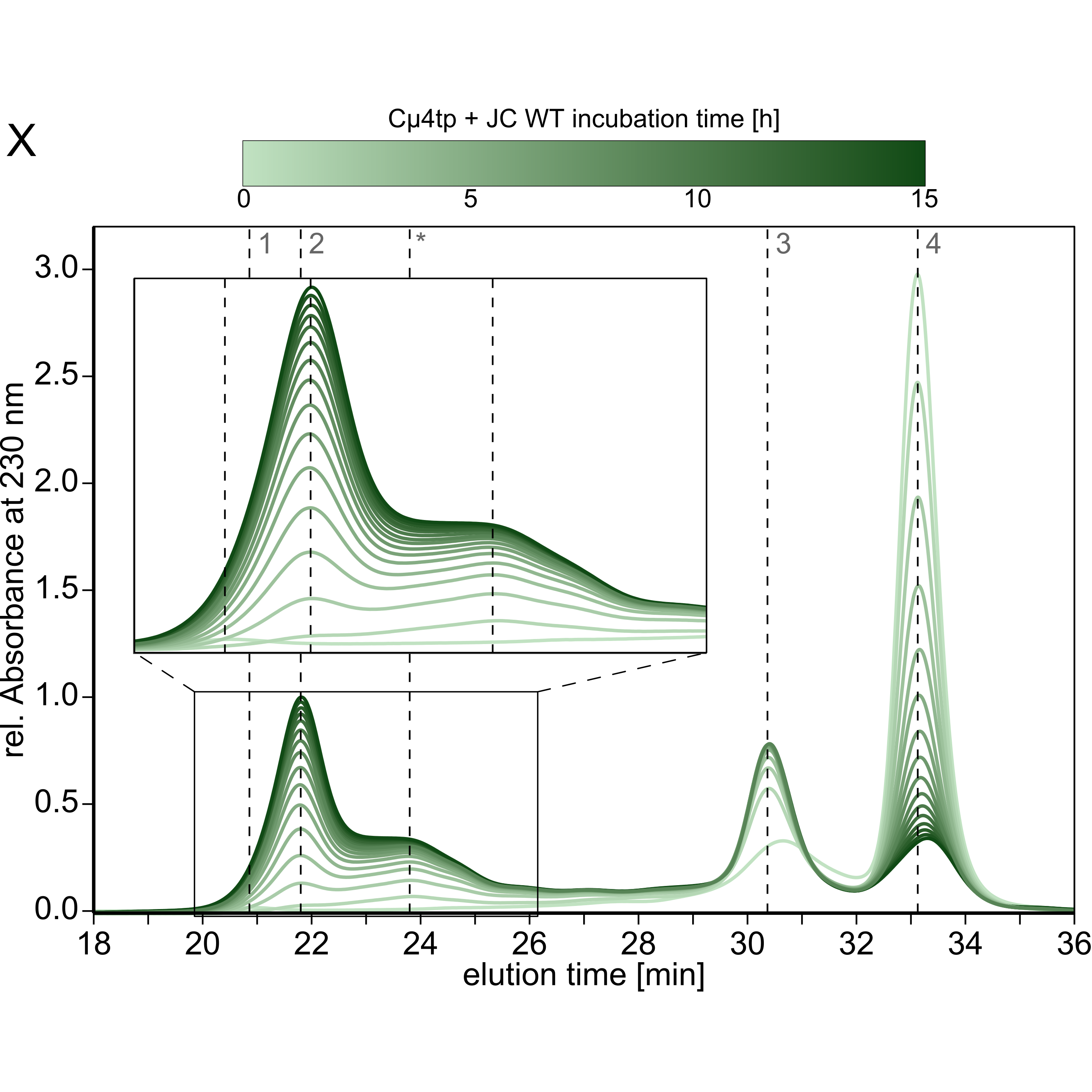

Supplement: Supplementary file 5 — Source data Fig. 3 [file 44318_2024_317_MOESM5_ESM.zip › Figure 3/3B/repeat 2 Cu4 + JC/Kinetics_2 with insert.png]

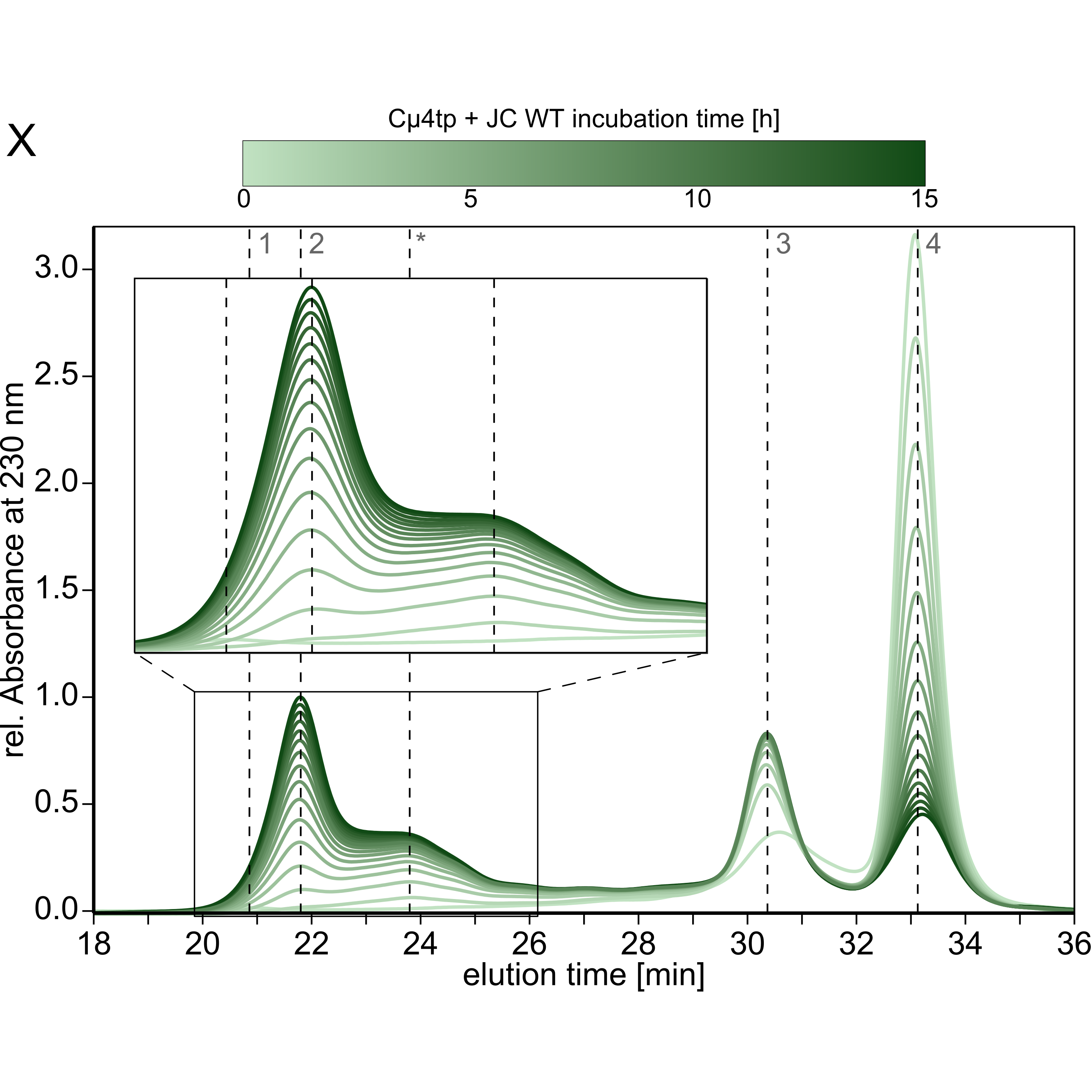

Supplement: Supplementary file 5 — Source data Fig. 3 [file 44318_2024_317_MOESM5_ESM.zip › Figure 3/3B/repeat 3 Cu4 + JC/Kinetics_3 with insert.png]

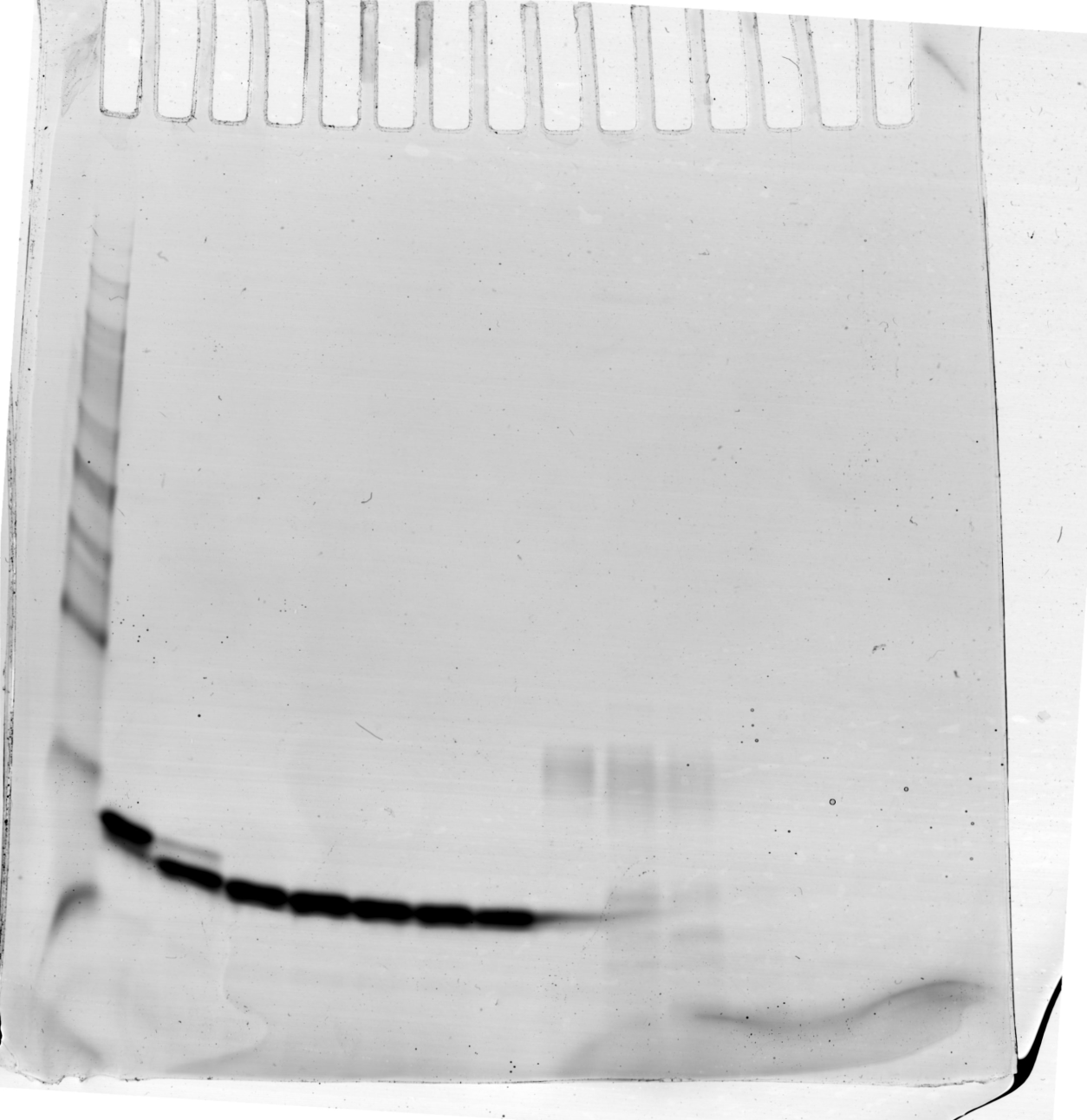

Supplement: Supplementary file 5 — Source data Fig. 3 [file 44318_2024_317_MOESM5_ESM.zip › Figure 3/3E/SDS PAGE 3E.png]

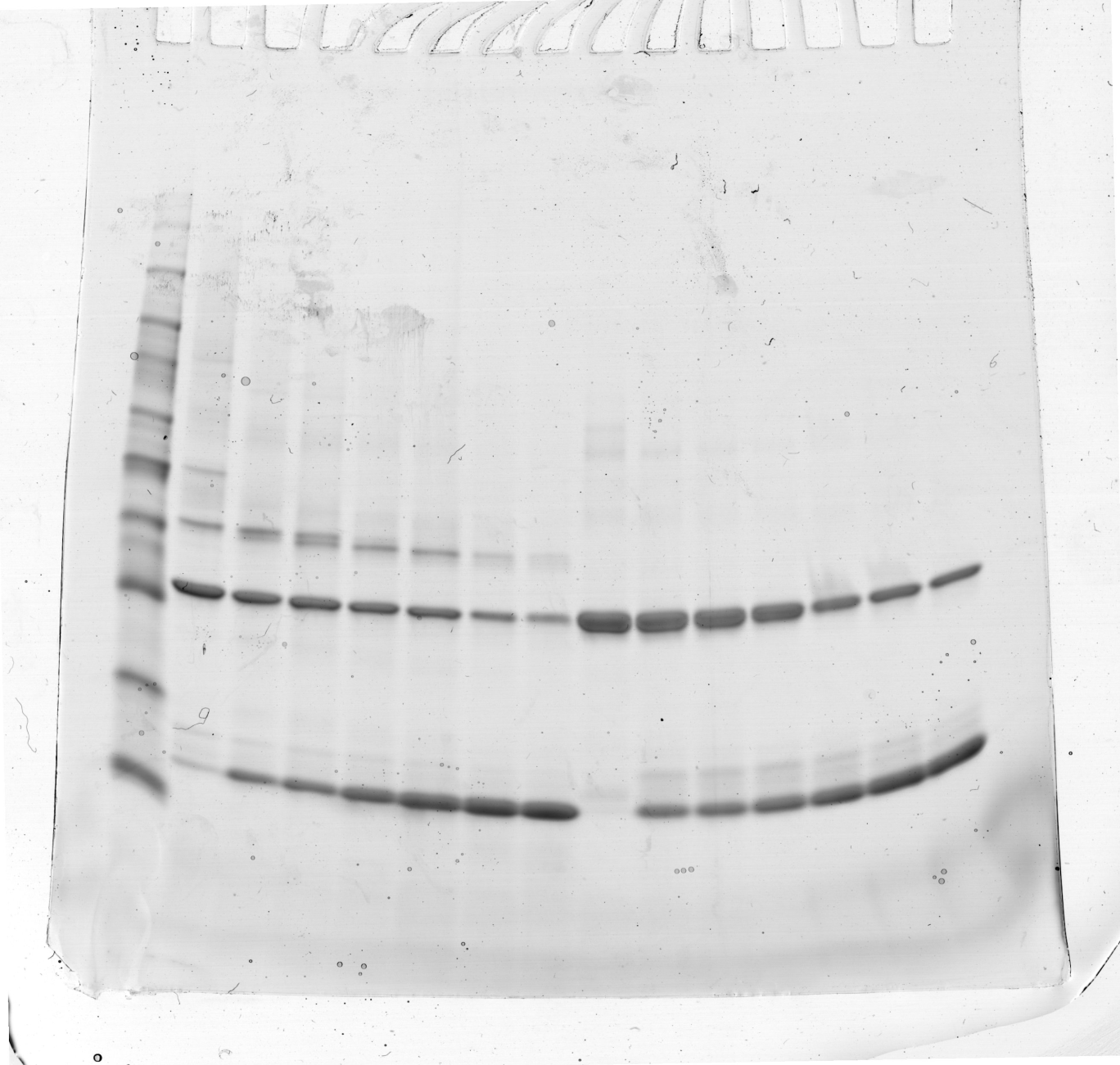

Supplement: Supplementary file 5 — Source data Fig. 3 [file 44318_2024_317_MOESM5_ESM.zip › Figure 3/3F/SDS PAGE 3F.png]

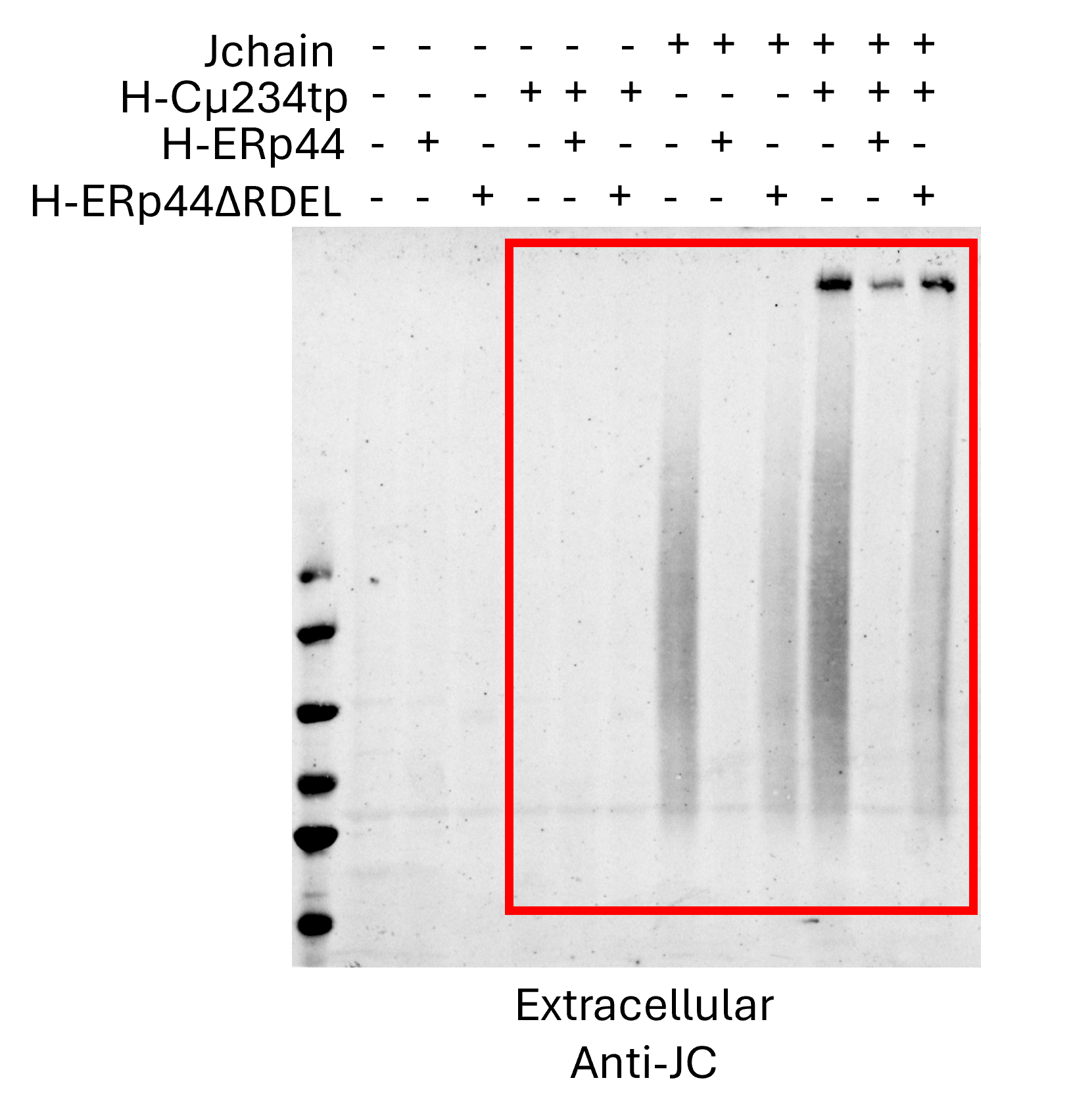

Supplement: Supplementary file 7 — Source data Fig. 5 [file 44318_2024_317_MOESM7_ESM.zip › Figure 5/5A/5A.tif]

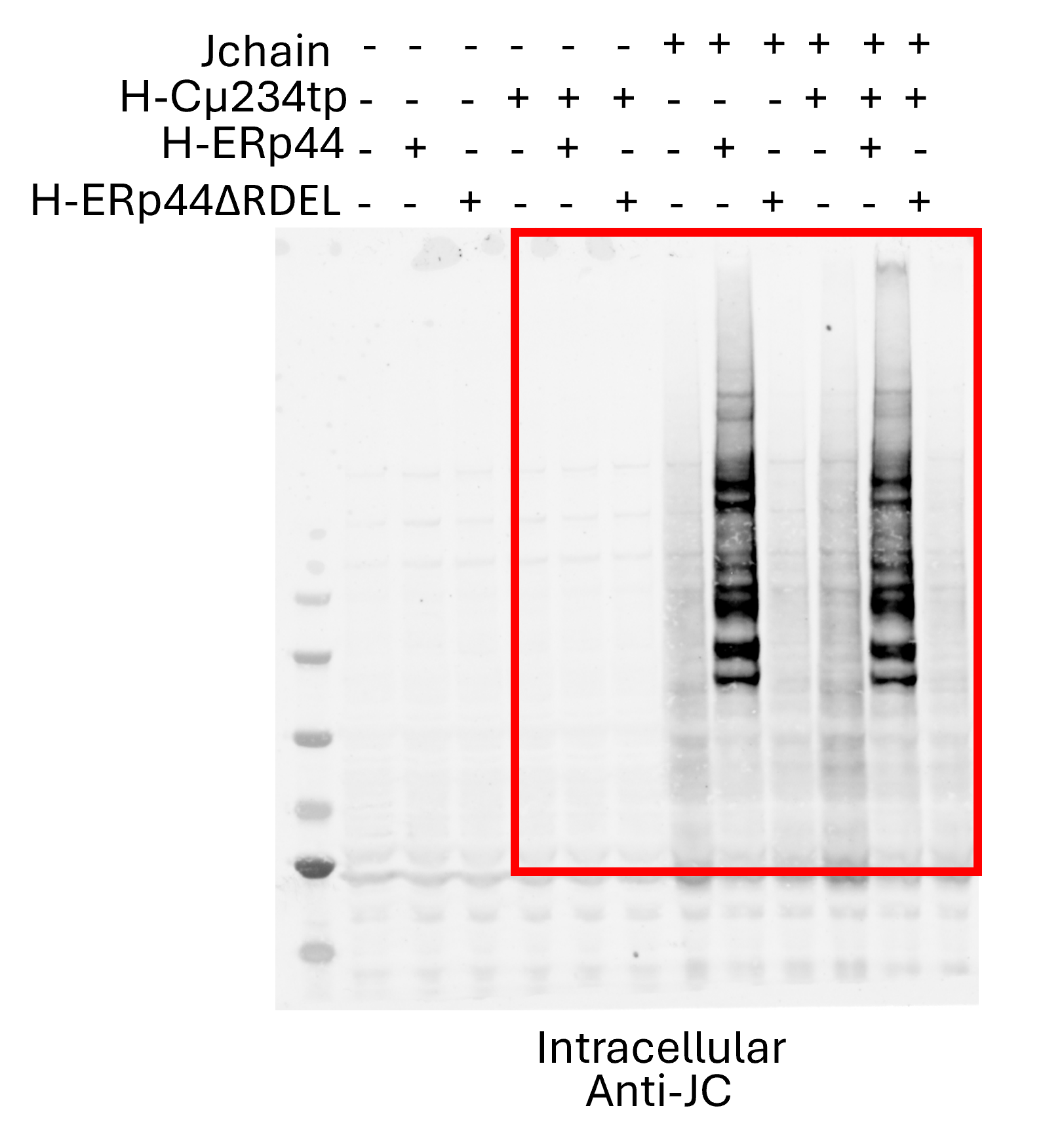

Supplement: Supplementary file 7 — Source data Fig. 5 [file 44318_2024_317_MOESM7_ESM.zip › Figure 5/5B/5B.tif]

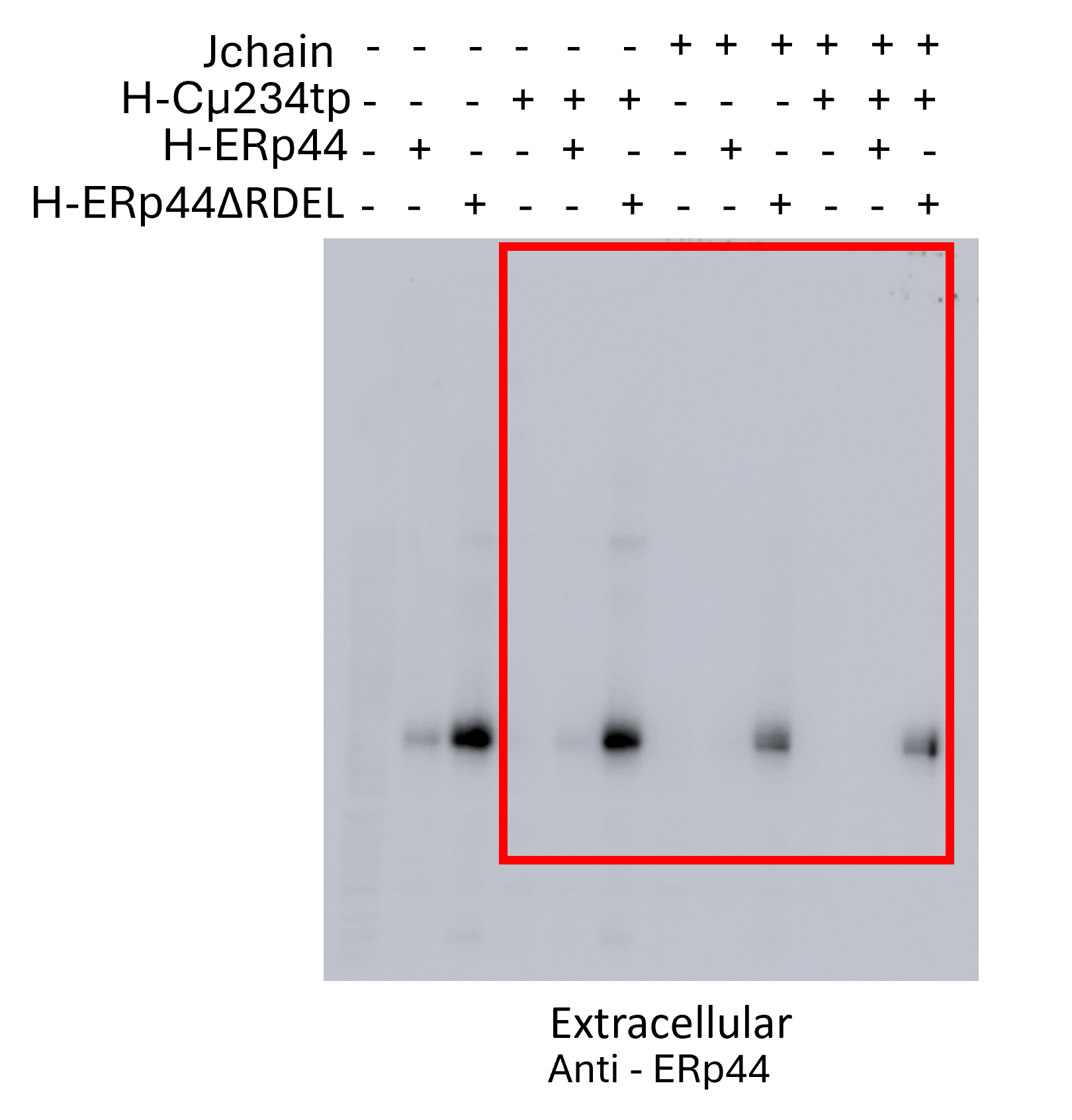

Supplement: Supplementary file 7 — Source data Fig. 5 [file 44318_2024_317_MOESM7_ESM.zip › Figure 5/5C/5C.tif]

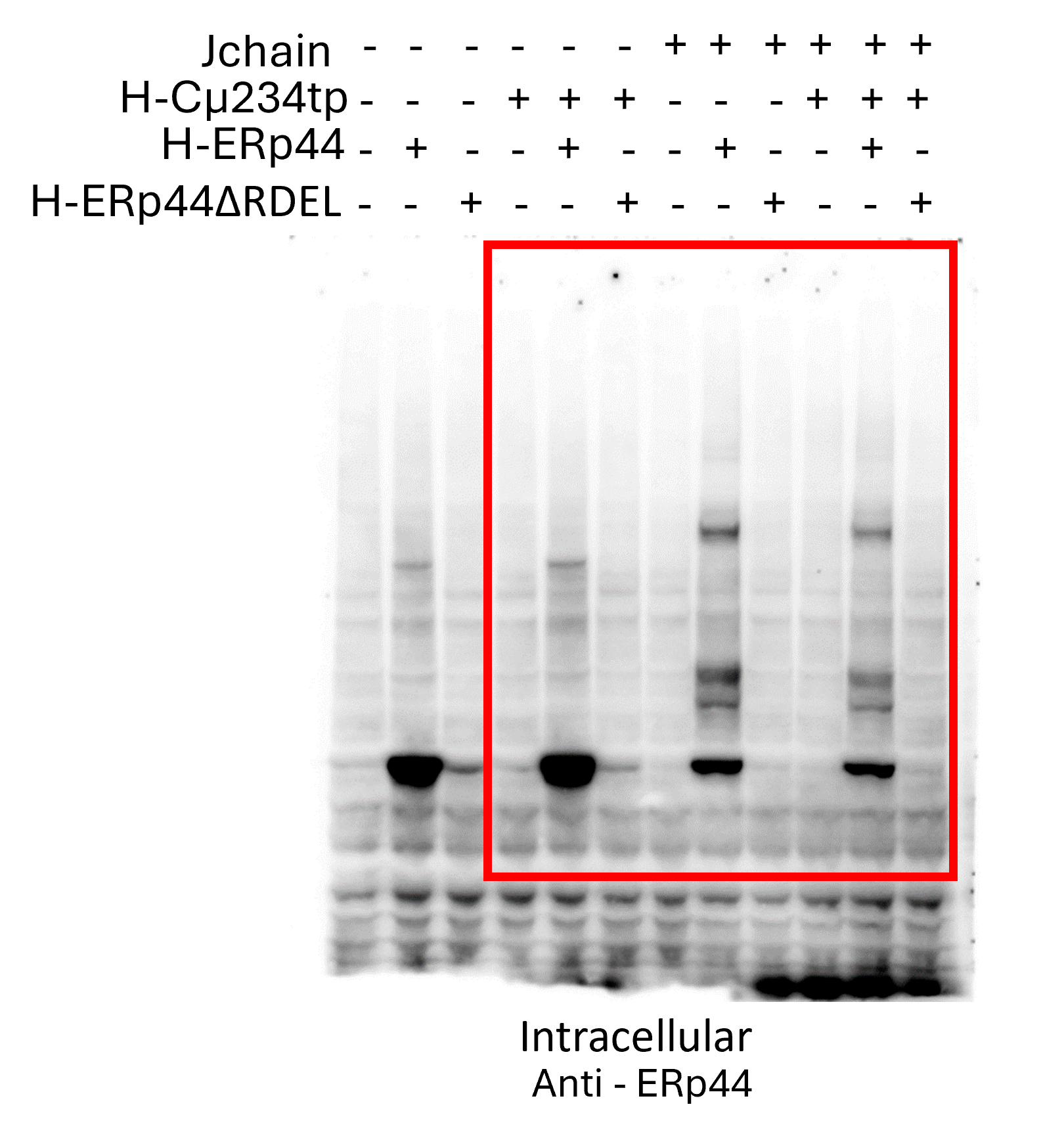

Supplement: Supplementary file 7 — Source data Fig. 5 [file 44318_2024_317_MOESM7_ESM.zip › Figure 5/5D/5D.tif]

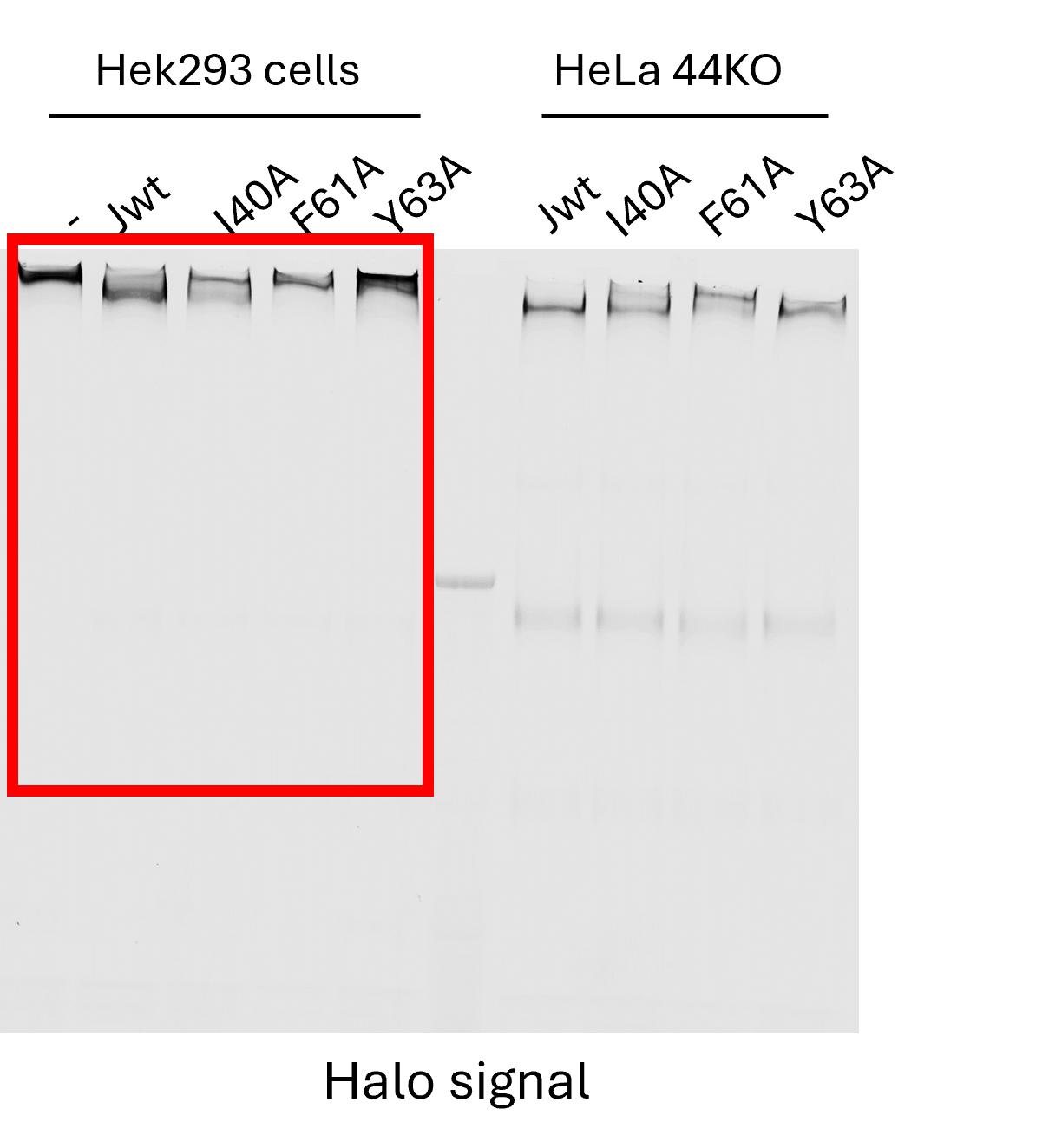

Supplement: Supplementary file 8 — Source data Fig. 6 [file 44318_2024_317_MOESM8_ESM.zip › Figure 6/6B/Halo signal extracellular.tif]

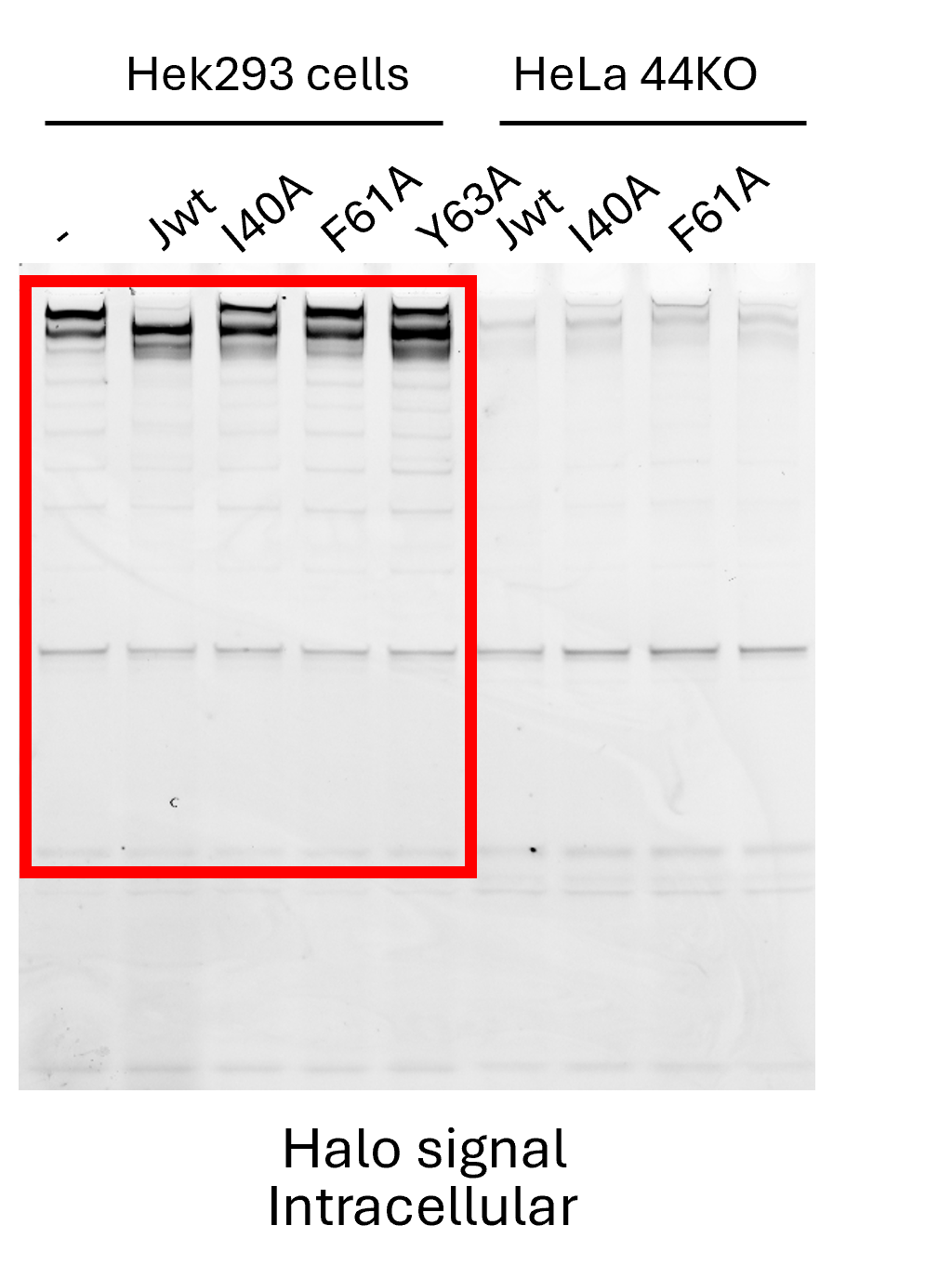

Supplement: Supplementary file 8 — Source data Fig. 6 [file 44318_2024_317_MOESM8_ESM.zip › Figure 6/6B/Halo signal intracellular.tif]

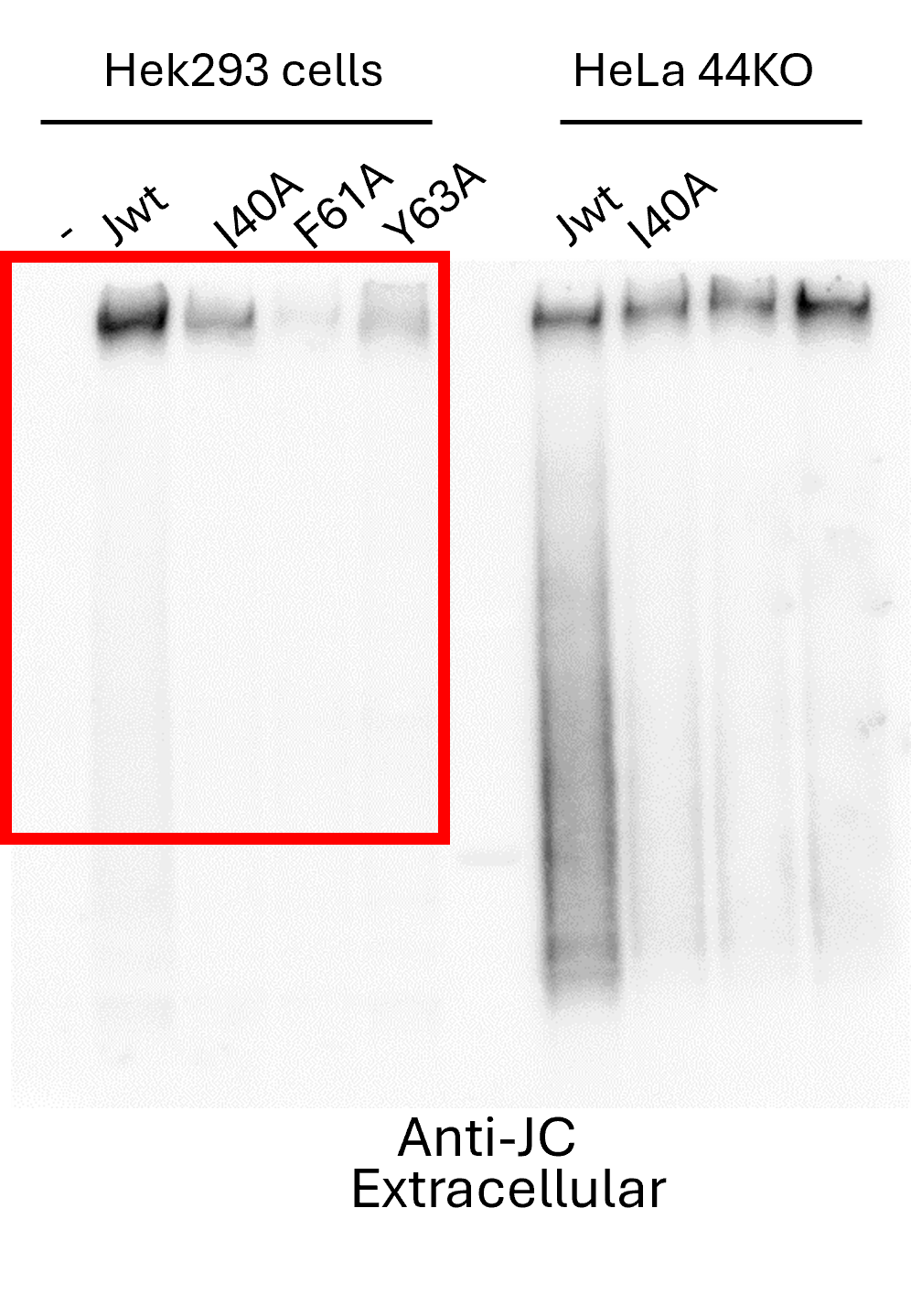

Supplement: Supplementary file 8 — Source data Fig. 6 [file 44318_2024_317_MOESM8_ESM.zip › Figure 6/6C/Western JC extracellular.tif]

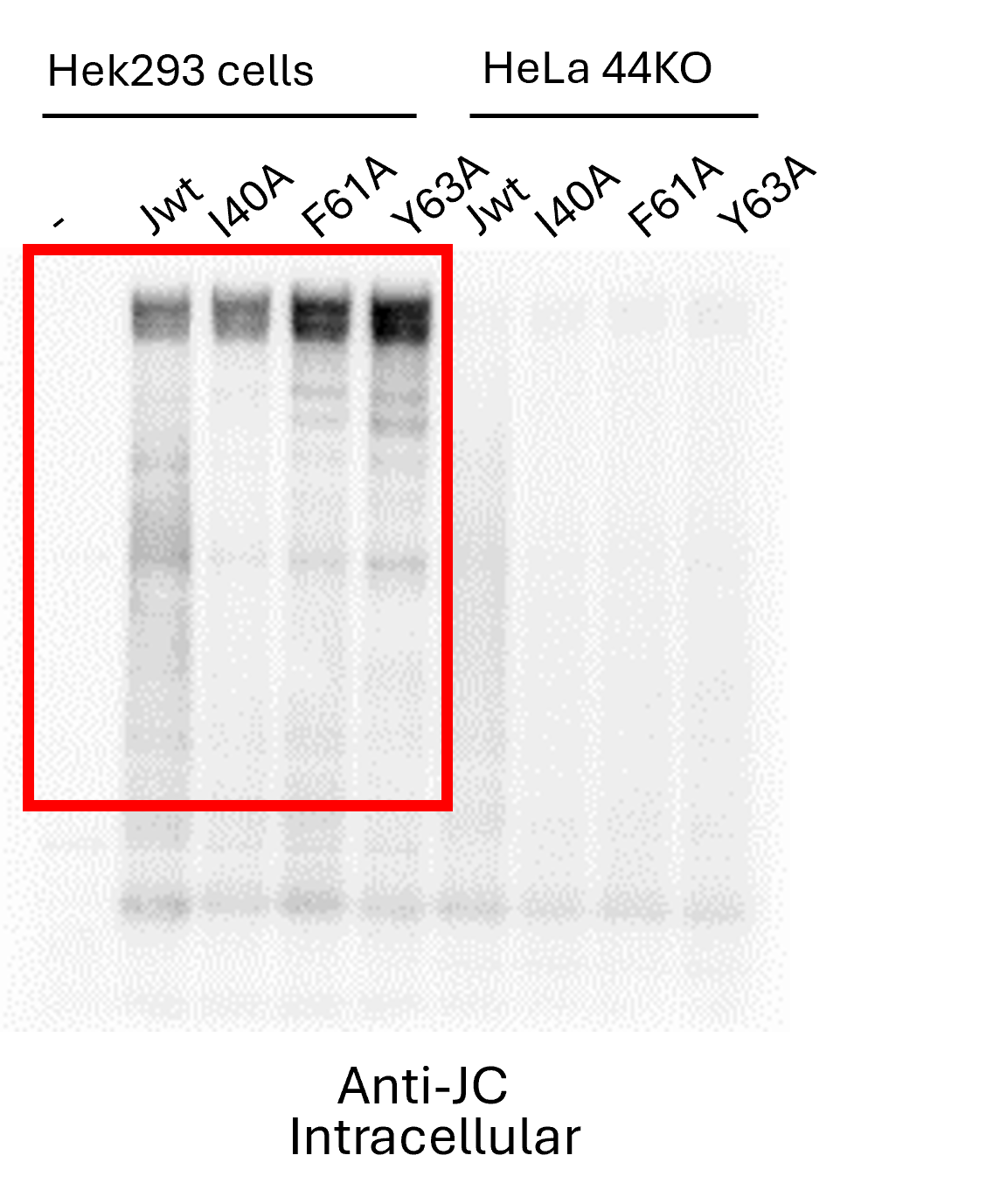

Supplement: Supplementary file 8 — Source data Fig. 6 [file 44318_2024_317_MOESM8_ESM.zip › Figure 6/6C/Western JC intracellular.tif]

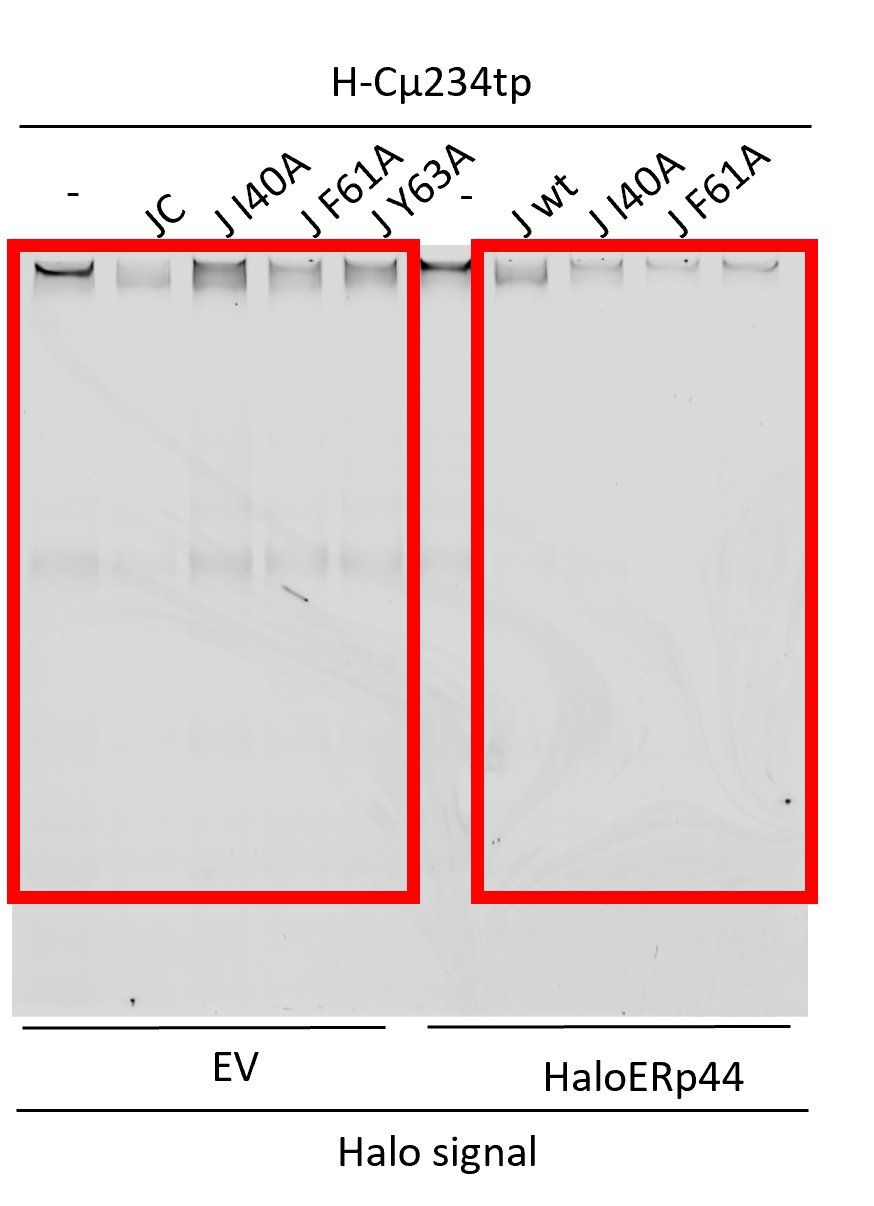

Supplement: Supplementary file 8 — Source data Fig. 6 [file 44318_2024_317_MOESM8_ESM.zip › Figure 6/6D/Halo signal.tif]

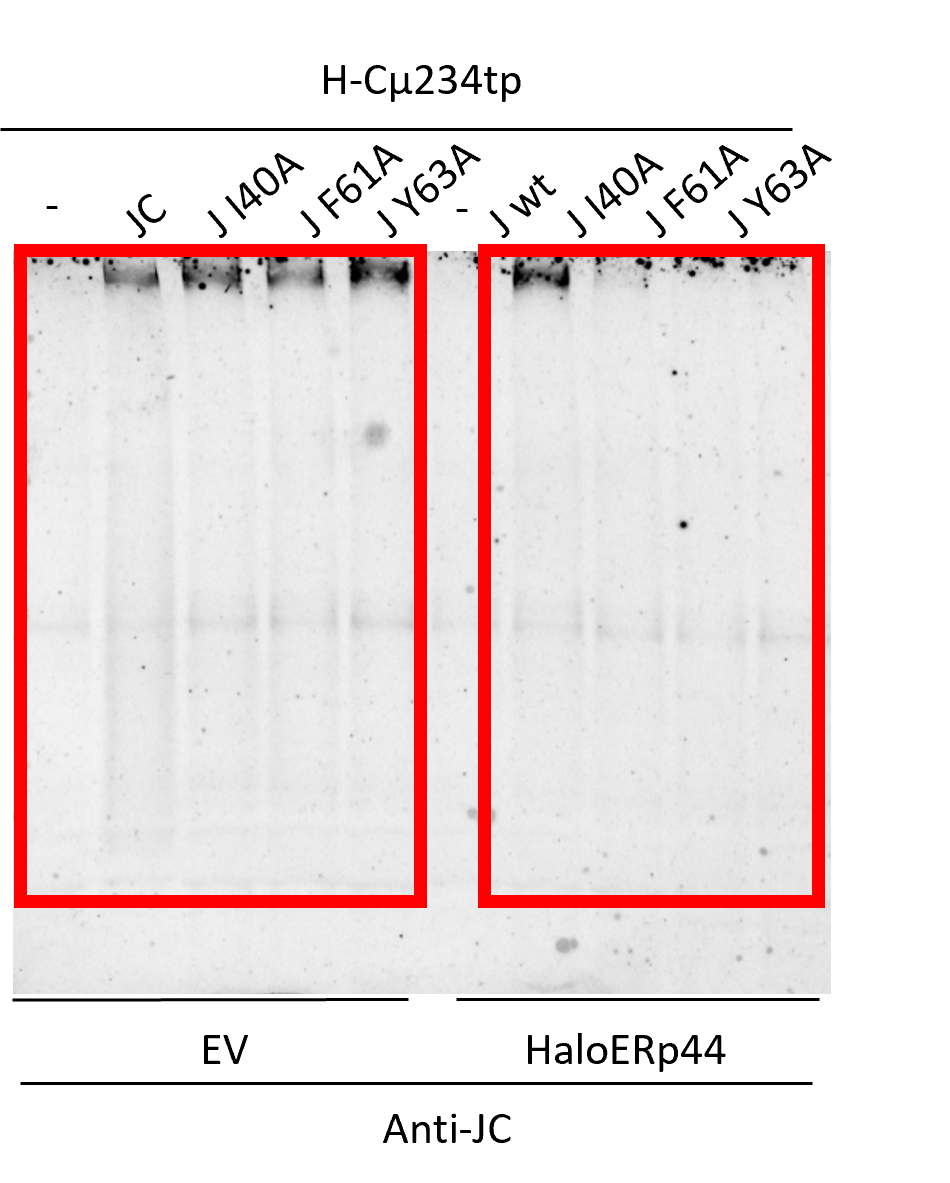

Supplement: Supplementary file 8 — Source data Fig. 6 [file 44318_2024_317_MOESM8_ESM.zip › Figure 6/6E/western JC.tif]

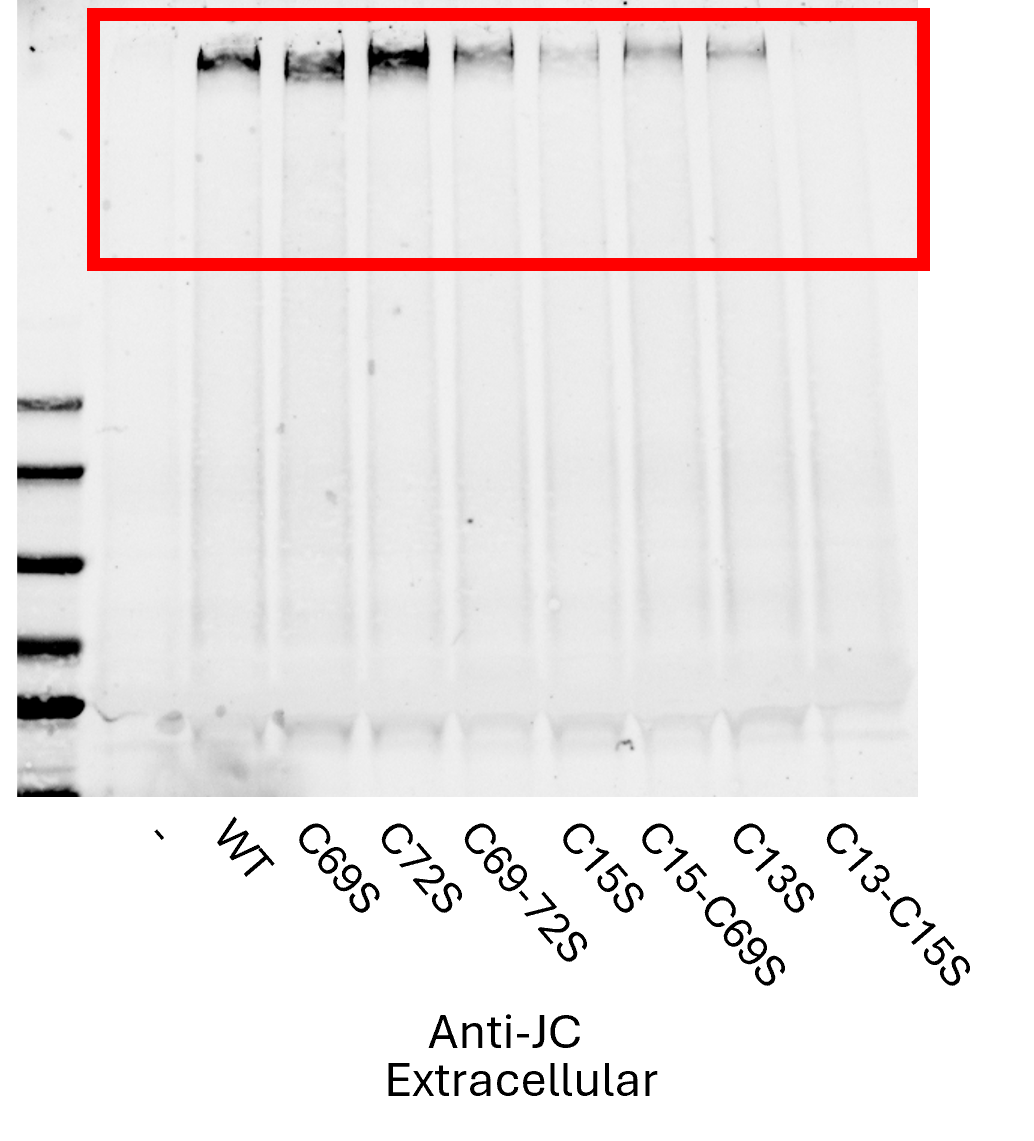

Supplement: Supplementary file 9 — Source data Fig. 7 [file 44318_2024_317_MOESM9_ESM.zip › Figure 7/7A/Western JC.tif]

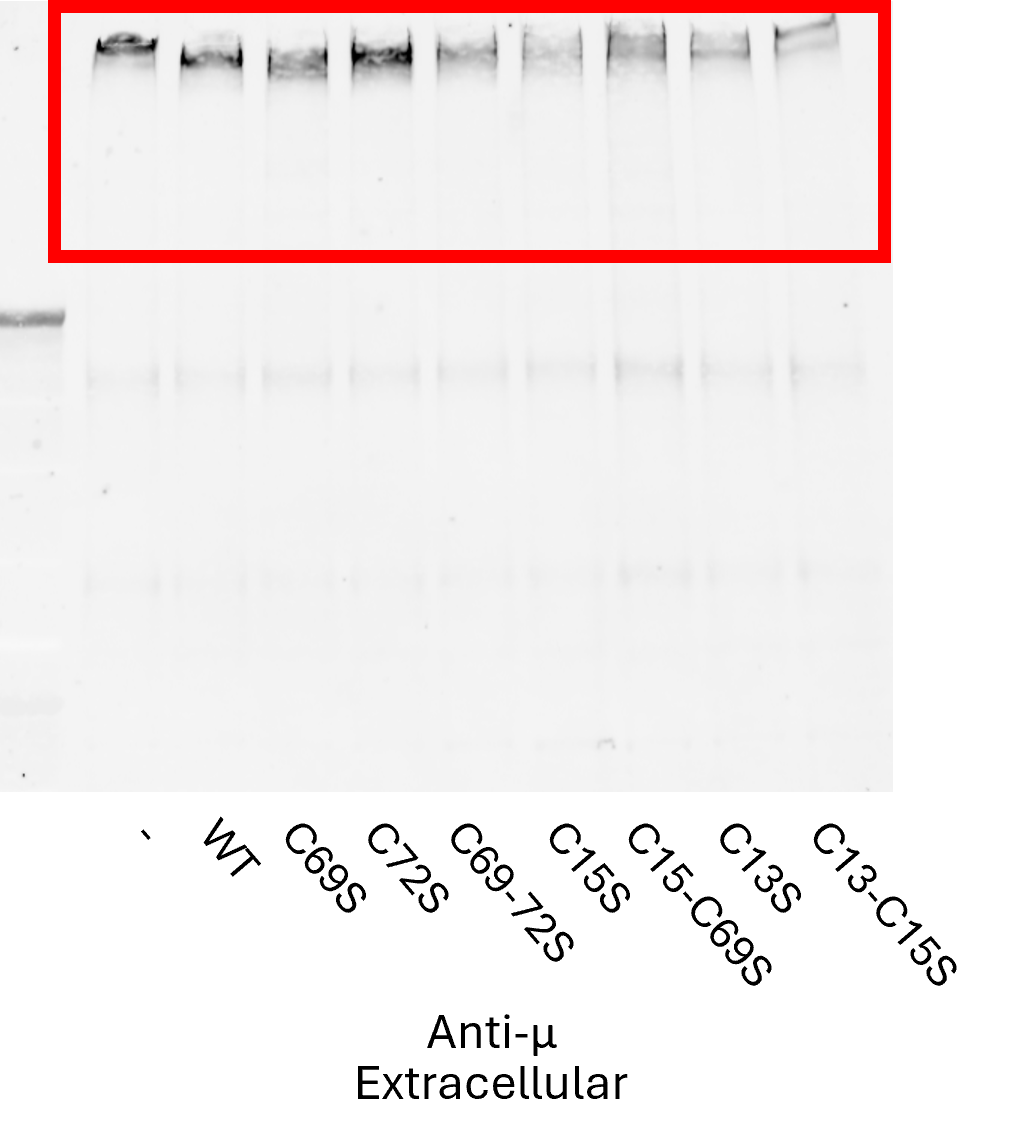

Supplement: Supplementary file 9 — Source data Fig. 7 [file 44318_2024_317_MOESM9_ESM.zip › Figure 7/7B/Western heavy chain.tif]
