# Supplementary material for: How J-chain ensures the assembly of immunoglobulin IgM pentamers
Source: EMBO J. 2024 Dec 4;44(2):505–33. doi: 10.1038/s44318-024-00317-9 (PMC11729874; doi:10.1038/s44318-024-00317-9)
Supplement: Supplementary file 10 — Expanded View Figures [file 44318_2024_317_MOESM10_ESM.pdf]

Expanded View Figures

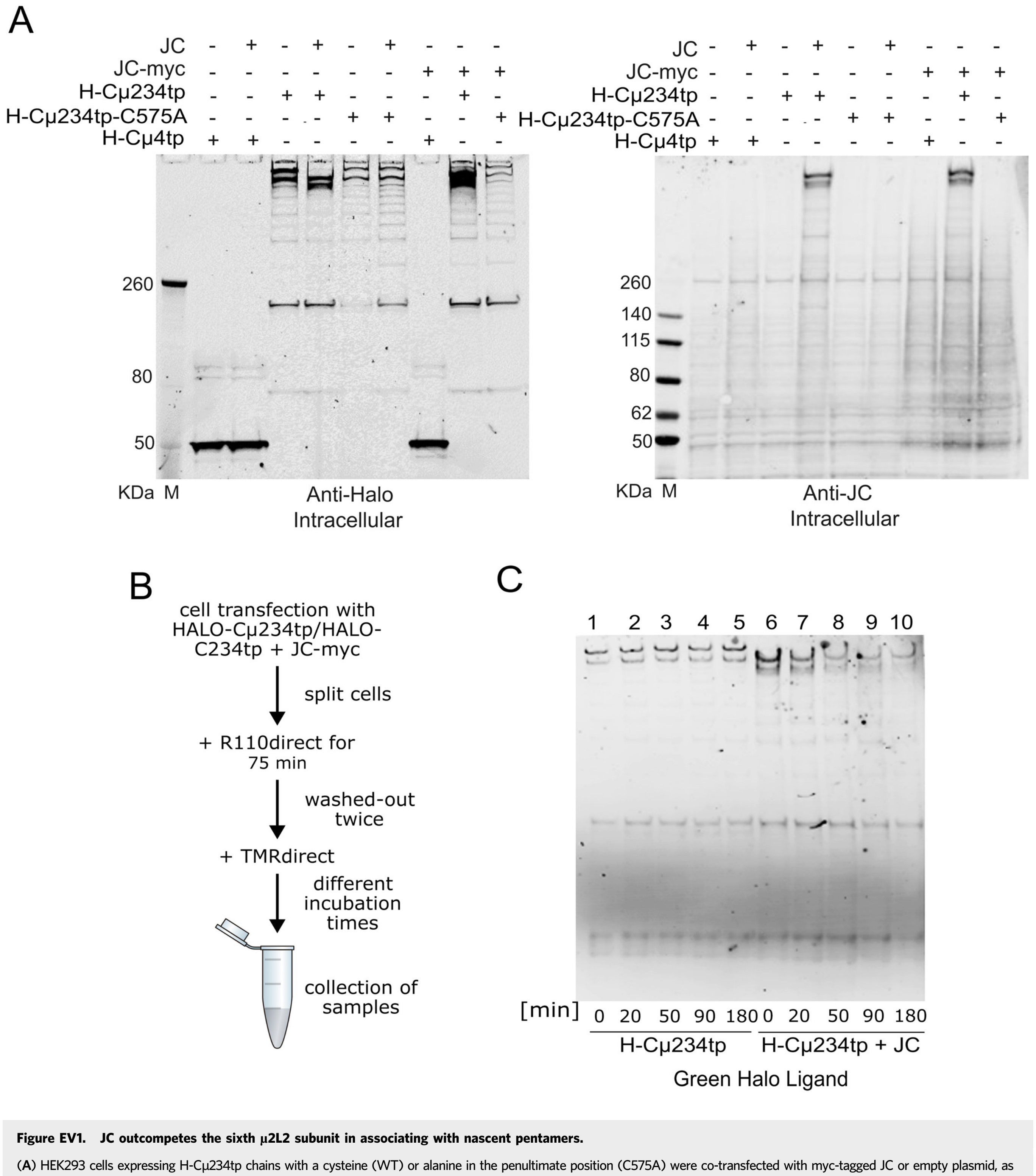

Figure EV1. JC outcompetes the sixth μ2L2 subunit in associating with nascent pentamers.

(**A**) HEK293 cells expressing H-Cμ234tp chains with a cysteine (WT) or alanine in the penultimate position (C575A) were co-transfected with myc-tagged JC or empty plasmid, as indicated. Aliquots of their lysates were resolved under nonreducing conditions and blots were decorated with Halo ligands (left panel) or anti-JC (right panel). (**B**). Halo time-course and kinetics of IgM assembly. As schematically summarized, HEK cells were co-transfected with H-Cμ234tp with or without JC-myc. Forty-eight hours after transfection, cells were incubated with green HALOtag fluorescent ligand (R110Direct, Promega) for 75 min, to label all preexisting molecules. Then cells were washed and incubated with red HALOtag fluorescent ligand (TMRDirect, Promega) for the indicated times (minutes). Cell lysates were analyzed electrophoretically (panel (**C**)) under nonreducing conditions, and the gel was directly analyzed for fluorescent R110 ligand. Note that hexamers are no longer detectable upon JC addition. JC do not favor the accumulation of dimers of H-Cμ234tp dimers, consistent with their preferential binding to nascent pentamers. As expected, H-Cμ234tp assembly species labeled before the addition of the red label tend to disappear with time.

© The Author(s)

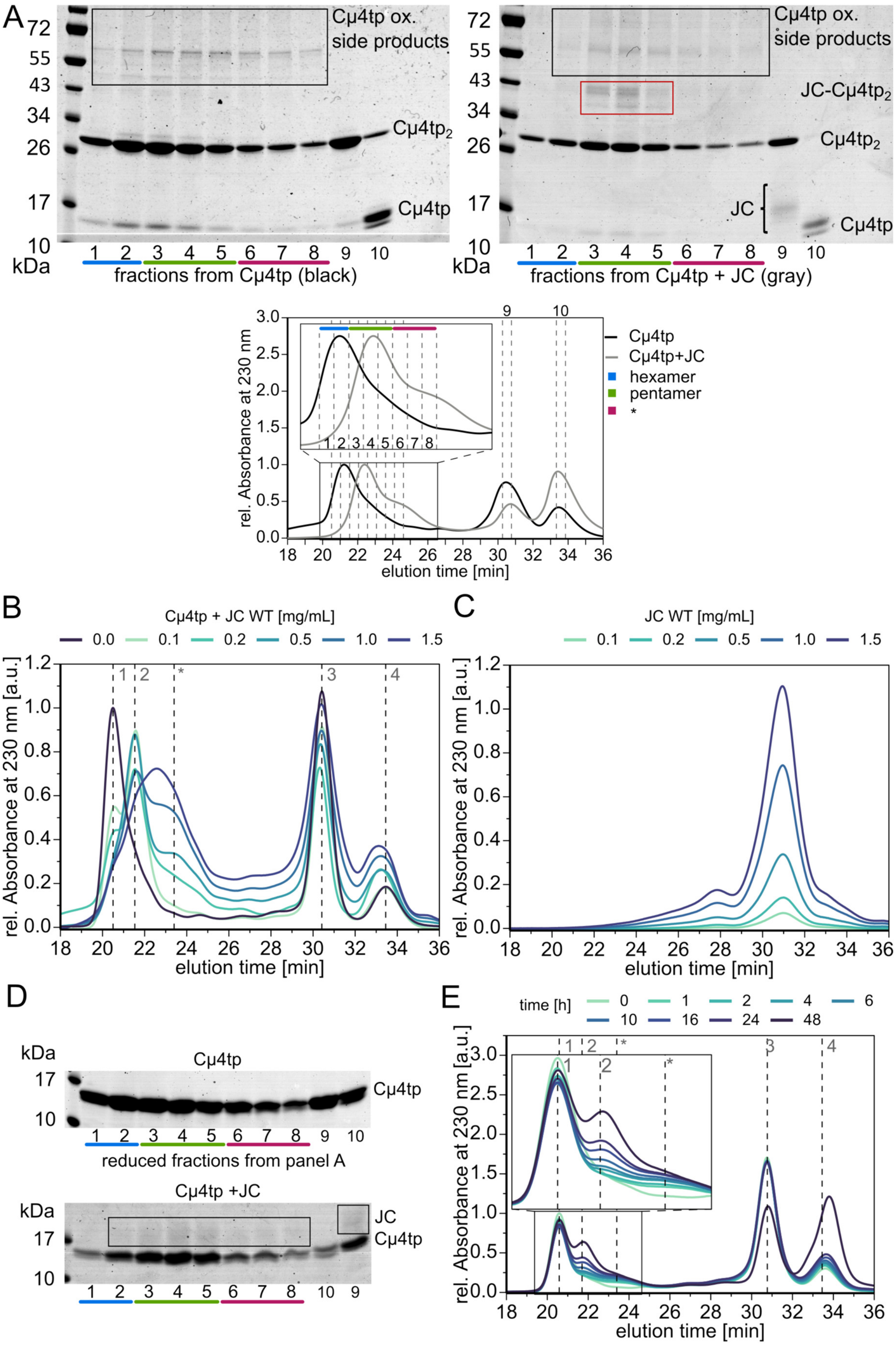
A
Cμ4tp ox. side products
Cμ4tp$_2$
Cμ4tp
JC-Cμ4tp$_2$
JC
kDa
fractions from Cμ4tp (black)
fractions from Cμ4tp + JC (gray)
rel. Absorbance at 230 nm
elution time [min]
Cμ4tp
Cμ4tp+JC
hexamer
pentamer
*
B
Cμ4tp + JC WT [mg/mL]
0.0 0.1 0.2 0.5 1.0 1.5
rel. Absorbance at 230 nm [a.u.]
elution time [min]
C
JC WT [mg/mL]
0.1 0.2 0.5 1.0 1.5
D
Cμ4tp
reduced fractions from panel A
Cμ4tp +JC
E
time [h]
0 1 2 4 6 10 16 24 48

Figure EV2. JC outcompete Cμ4tp$_2$ subunits to complete stable hexamers.

(**A**) Composition of the fractions obtained from preparative HPLC-SE. Aliquots of the high molecular peaks obtained by incubating Cμ4tp (1 mg/mL) with (black line) or without JC (0.5 mg/mL) (gray line) were applied to SE-HPLC and 250 μL and the indicated fractions (lower panel) analyzed by SDS-PAGE under nonreducing conditions (top two panels) or protease sensitivity assays (panel **D**). Fractions 1–2 (blue), correspond to hexamers, fractions 3–5 (green) to pentamer, fractions 6-8 (red) to the shoulder peak *, whilst fractions 9 and 10 contain JC+Cμ4tp dimers, or mainly Cμ4 monomers, respectively. As expected, species overlap to some degree. In the presence of JC, additional bands corresponding to a Cμ4tp$_2$-JC species are visible on SDS-PAGE (left panel, red box) in the pentamer and to a lesser extent in the * fractions. These bands are absent when Cμ4tp incubated alone. Cμ4tp on its own forms hexamers, which disassemble to dimers on SDS-PAGE (right panel). Some Cμ4tp oxidation side product bands (both panels, black boxes) are evident in the upper parts of both gels. These side products might contribute to the formation of the * shoulder. (**B**, **C**) JC outcompetes the sixth Cμ4tp$_2$ subunit in vitro. Increasing the amount of JC (from 0 to 1.5 mg/ml) added to 1 mg/ml Cμ4, progressively inhibits the formation of (Cμ4tp$_2$)$_6$ "hexamers" in favor of (Cμ4tp$_2$)$_5$-JC "pentamers" (panel **B**). At their highest concentration, a broad shoulder appears that presumably corresponds to aberrant assemblies in various stoichiometries. In the absence of Cμ4tp (panel **C**), most JC accumulates as a broad peak. A higher molecular weight shoulder was also observed, indicative of multimeric JC species, consistent with the smeary appearance of JC in cells (see Figs. 5 and EV3, panels **A**, **B**). (**D**) Reducing SDS-PAGE of the fractions shown in panel A confirms that JC is covalently linked to Cμ4tp in the oligomeric assemblies. All chromatograms were recorded thrice. (**E**) JC cannot be inserted in preformed (Cμ4tp$_2$)$_6$ hexamers. Preformed (Cμ4tp$_2$)$_6$ hexamers were incubated with 0.5 mg/mL amounts of JC for 48 h at room temperature and finally analyzed by SEC. The size of peak 1 remains constant, indicating that once formed, hexamers are rather stable. The increase in Peak 2 reflects the formation of pentamers during the incubation.

© The Author(s)

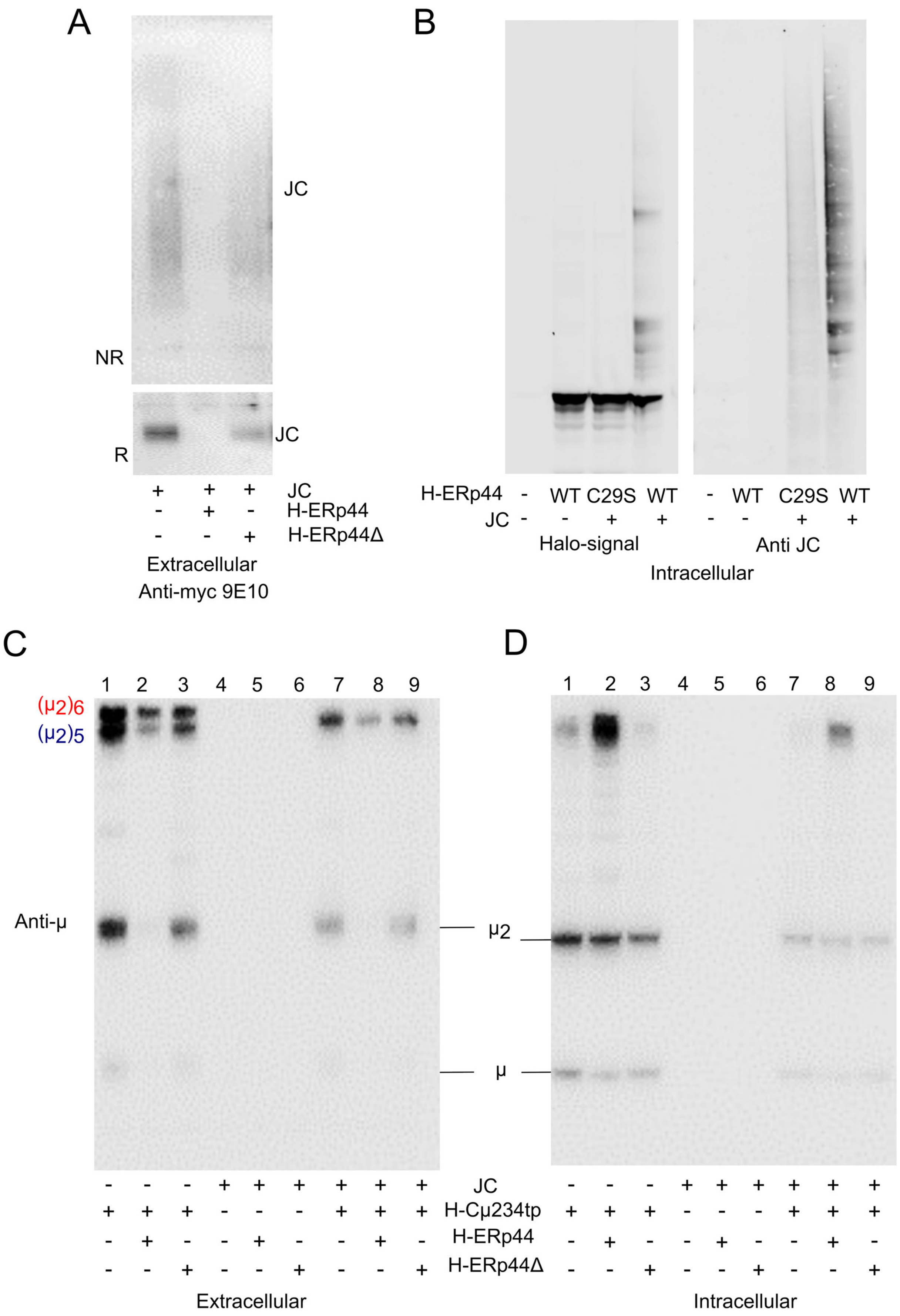
A
JC
NR
R
JC
+ + + JC
- + - H-ERp44
- - + H-ERp44Δ
Extracellular
Anti-myc 9E10
B
H-ERp44 - WT C29S WT
JC - - + +
Halo-signal
- WT C29S WT
- - + +
Anti JC
Intracellular
C
1 2 3 4 5 6 7 8 9
(μ2)6
(μ2)5
Anti-μ
μ2
μ
JC
H-Cμ234tp
H-ERp44
H-ERp44Δ
Extracellular
D
1 2 3 4 5 6 7 8 9
Intracellular

 © The Author(s)

◀ **Figure EV3. ERp44^{KO} cells secrete JC rich in heterogeneous, non-native disulfides.**

(**A**) When aliquots from the supernatants of ERp44^{KO} transfectants expressing myc-tagged JC are resolved electrophoretically under nonreducing conditions, anti-myc antibodies decorate a continuous smear. In contrast, DTT reduces the smear, and JC-myc chains accumulate as a 20–25 kDa band. Without alkylation, some intra-chain disulfide bonds may form during electrophoresis. Expression of WT ERp44 (center lane), but not of its ΔRDEL mutant (right lane), inhibits JC-myc secretion. All chromatograms were recorded thrice. (**B**) ERp44 binds JC via its C29. HeLa-ERp44^{KO} cells were transfected as indicated, and their lysates were run under nonreducing conditions and blotted with Halo ligand (left) or anti-JC. H-ERp44 forms many abundant complexes with JC that are not formed by a mutant lacking C29. Not all bands detected by anti-JC contain H-ERp44 (right panel). These data confirm that ERp44 efficiently retains JC through C29-dependent reversible disulfide bonds. (**C**, **D**) ERp44 prevents the secretion of unpolymerized IgM. HeLa-ERp44^{KO} cells were transfected as indicated, and aliquots from their supernatants (**C**) or lysates (**D**) resolved under nonreducing conditions and decorated with anti-μ antibodies. ERp44^{KO} HeLa cells secrete abundant H-Cμ234tp$_2$ subunits (**D**, lane 1). Co-expression of wild-type ERp44 restores retention of these incomplete subunits and promotes polymerization (panel **D**, compare lanes 1 and 2). H-ERp44 expression boosts also the formation of JC-containing pentamers (panel **D**, lanes 7-8), dampening the secretion of polymers, irrespective of the presence of JC (panel **C** lanes 7-8). H-ERp44Δ lacks the RDEL motif.

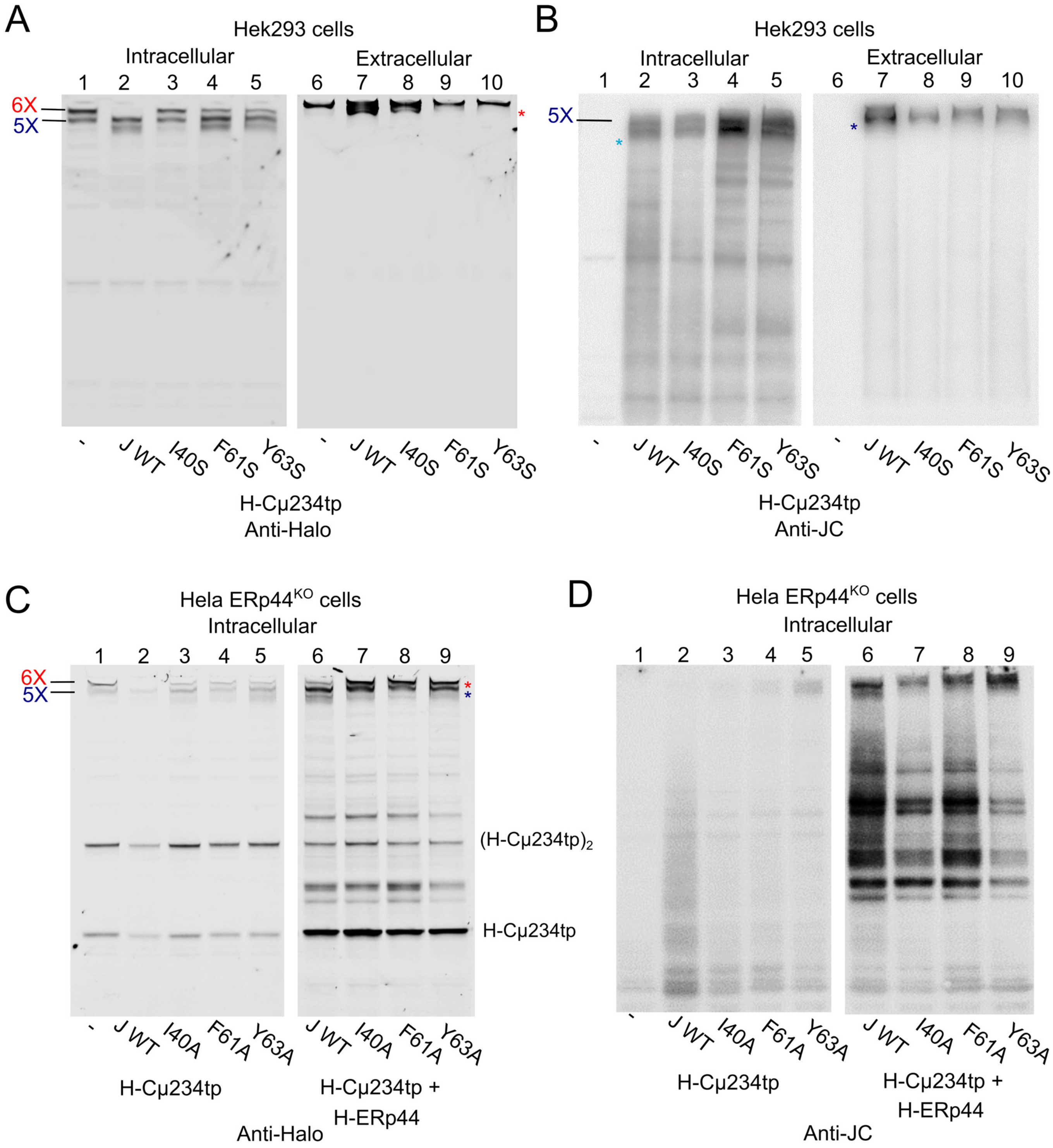
A
Hek293 cells
Intracellular
Extracellular
1 2 3 4 5
6 7 8 9 10
6X
5X
*
- J WT I40S F61S Y63S - J WT I40S F61S Y63S
H-Cμ234tp
Anti-Halo
B
Hek293 cells
Intracellular
Extracellular
1 2 3 4 5
6 7 8 9 10
5X
*
*
- J WT I40S F61S Y63S - J WT I40S F61S Y63S
H-Cμ234tp
Anti-JC
C
Hela ERp44KO cells
Intracellular
1 2 3 4 5
6 7 8 9
6X
5X
*
*
(H-Cμ234tp)2
H-Cμ234tp
- J WT I40A F61A Y63A J WT I40A F61A Y63A
H-Cμ234tp
H-Cμ234tp +
H-ERp44
Anti-Halo
D
Hela ERp44KO cells
Intracellular
1 2 3 4 5
6 7 8 9
- J WT I40A F61A Y63A J WT I40A F61A Y63A
H-Cμ234tp
H-Cμ234tp +
H-ERp44
Anti-JC

© The Author(s)

Figure EV4. Conserved hydrophobic residues drive assembly of secretion-competent IgM pentamers.

(**A**, **B**) Essential roles of hydrophobic interactions. Replacing I40, F61, and Y63 with serine yields phenotypes similar or slightly more evident than alanine substitutions. Note that the three mutants are poorly inserted into secretion-competent pentamers (dark blue asterisk). As a result, hexamers (red asterisk) remain the most abundant intracellular species recognized by Halo ligands, and the sole one to be abundantly secreted by cells expressing ERp44. An uncharacterized form is retained intracellularly (light blue asterisk). Anti-JC antibodies intensely decorate F61S and Y63S in polymers accumulating intracellularly (lanes 4-5 panel **B**), but these are poorly secreted (lanes 9-10). Consequently, virtually only hexamers are detected by Halo ligands in the supernatants (lanes 9-10 panel **A**). (**C**, **D**). ERp44 binds JC as well as unpolymerized IgM intermediates. The lysates of $ERp44^{KO}$ cells expressing H-Cμ234tp, rescued with or without WT H-ERp44, were decorated with Halo ligands or anti-JC. As expected, overexpression of ERp44 prevents JC and H-Cμ234tp secretion, increasing their intracellular accumulation. The abundance of anti-JC reactive covalent complexes in lanes 6–9 (panel **D**) indicates that ERp44 has a high affinity for JC.

© The Author(s)

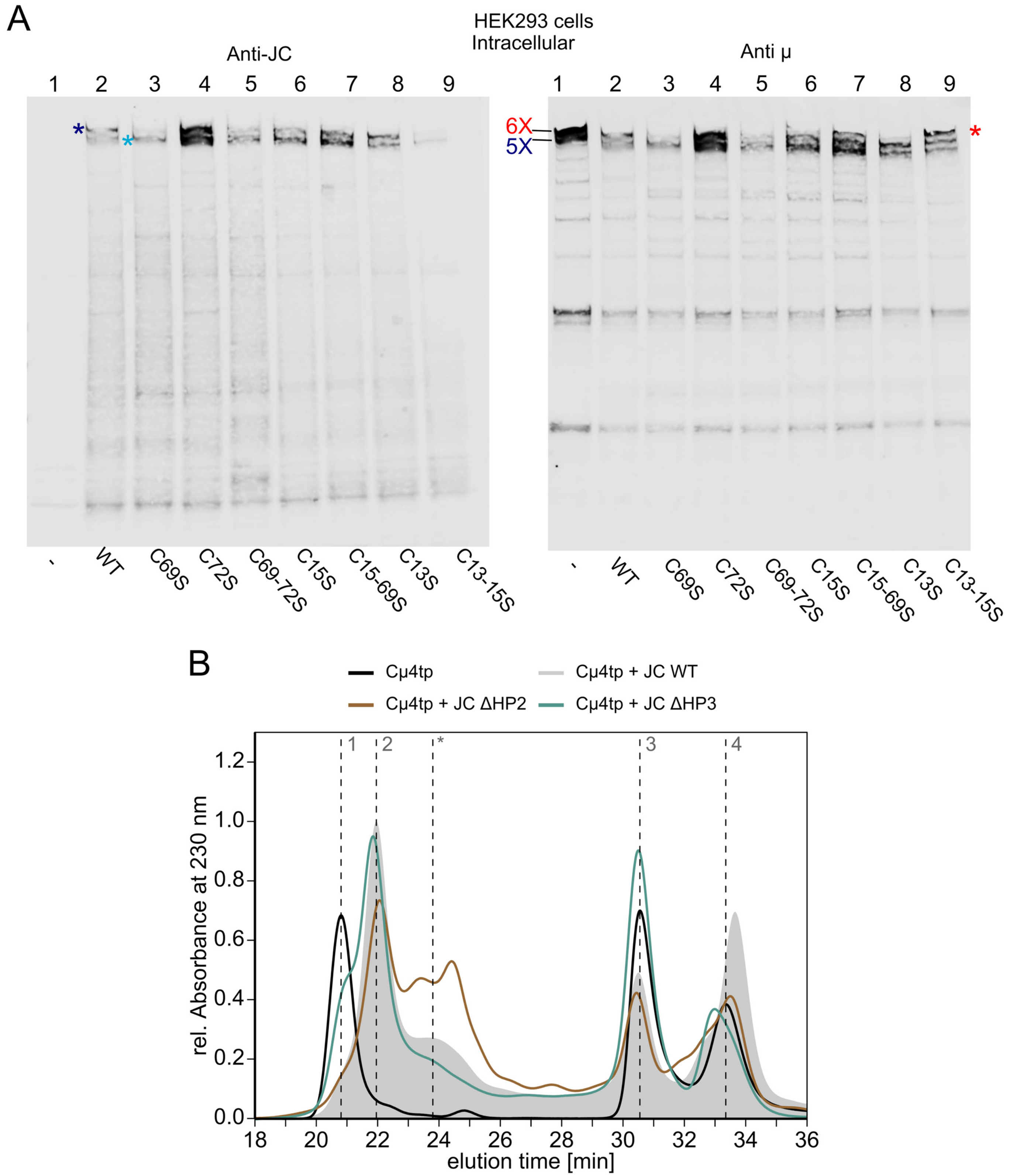
A
HEK293 cells
Intracellular
Anti-JC
Anti μ
1 2 3 4 5 6 7 8 9
6X
5X
- WT C69S C72S C69-72S C15S C15-69S C13S C13-15S
B
Cμ4tp
Cμ4tp + JC WT
Cμ4tp + JC ΔHP2
Cμ4tp + JC ΔHP3
rel. Absorbance at 230 nm
elution time [min]

 © The Author(s)

Figure EV5. Reactivity of the JC cysteine mutants in cellula and in vitro.

(**A**) Pivotal role of C13 and C15. The lysates of HEK293 transfectants co-expressing H-Cμ234tp and the indicated JC mutants were resolved under nonreducing conditions and blots sequentially decorated with anti-JC and anti-μ, as indicated. Notably, the C13-15S mutant fails to bind IgM (lane 9) and forms mainly hexamers (red asterisk) detected with anti- μ, whilst the other mutants do bind IgM (dark blue asterisk) although with different efficiencies. An uncharacterized form is retained intracellularly (light blue asterisk). (**B**) The C-terminal part of JC is not essential for pentamer binding. SEC profiles on a Superdex 200 increase 10/300 GL in PBS. Cμ4tp (1 mg/mL) was incubated in vitro with WT or mutant JC (0.5 mg/mL) for 24 h at RT. Whilst the deletion of hairpin 3 and its flanking cysteines C110 and C135 (ΔHP3, turquoise) does not alter the SEC elution pattern (compare the blue trace with the gray pattern corresponding to WT JC), removal of hairpin 2 and the cysteines C72 and C92 (ΔHP2, brown trace) severely inhibits the formation of pentamers in vitro. These findings show that C110 and C135 are not vital for the formation of IgM pentamers. All chromatograms were recorded thrice.

© The Author(s)
